# Supplementary material for: AAV-miR-204 Protects from Retinal Degeneration by Attenuation of Microglia Activation and Photoreceptor Cell Death
Source: Mol Ther Nucleic Acids. 2019 Nov 18;19:144–56. doi: 10.1016/j.omtn.2019.11.005 (PMC6920266; doi:10.1016/j.omtn.2019.11.005)
Supplement: Document S1. Figures S1–S8 [file mmc1.pdf]

OMTN, Volume *19*

## Supplemental Information

# AAV-miR-204 Protects from Retinal Degeneration by Attenuation of Microglia Activation and Photoreceptor Cell Death

**Marianthi Karali, Irene Guadagnino, Elena Marrocco, Rossella De Cegli, Annamaria Carissimo, Mariateresa Pizzo, Simona Casarosa, Ivan Conte, Enrico Maria Surace, and Sandro Banfi**

# Figure S1

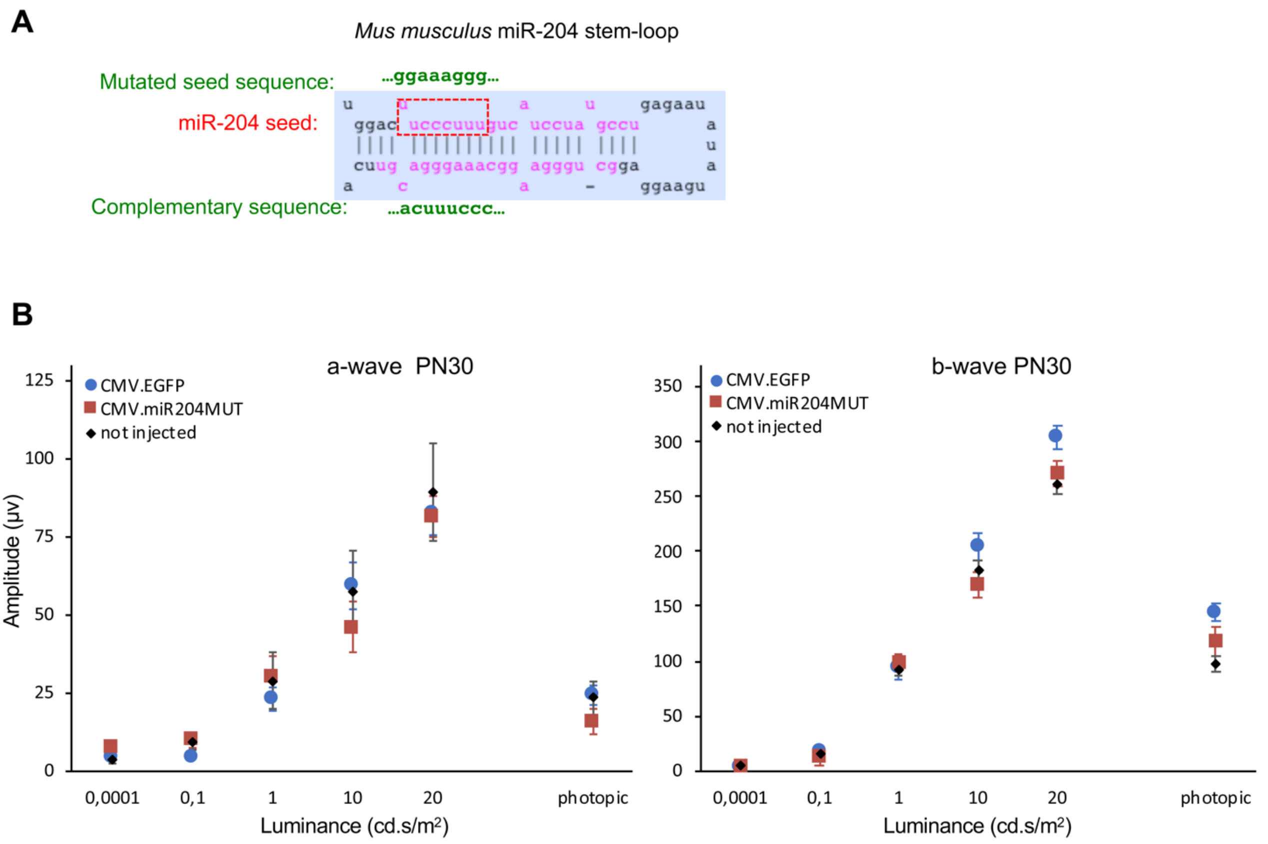


**Figure S1. ERG analysis of *RHO*-P347S transgenic mice following AAV-mediated delivery of control vectors used in this study at PN4.**

**(A)** Sequence of the miR-204 stem-loop in *Mus musculus* (blue box). The sequence of the mature miR-204-5p (upper strand) and miR-204-3p (lower strand) is in purple. The 'seed' region of miR-204-5p is in a red frame. The sequence introduced by site-directed mutagenesis to generate the AAV.CMV.miR204MUT control vector is in green.

**(B)** A-wave and b-wave ERG responses of *RHO*-P347S mice injected with AAV.CMV.EGFP (n=26) and AAV.CMV.miR204MUT (n=9) at PN4. Responses of non-injected eyes are shown in black. ERGs were recorded at PN30. Both control vectors elicited similar ERG responses and did not show significant differences compared to non-injected *RHO*-P347S eyes.

## Figure S2

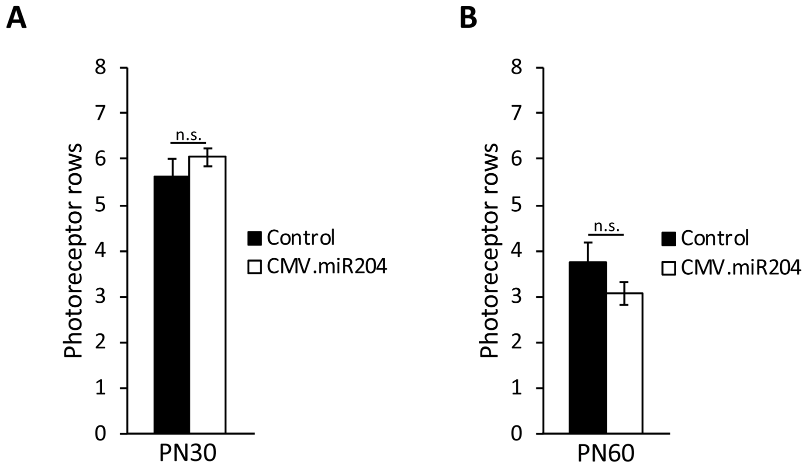


**Figure S2. ONL thickness in injected *RHO*-P347S eyes.**
Plots show the average number of photoreceptor nuclei rows at PN30 **(A)** and PN60 **(B)** in miR-204 treated *RHO*-P347S eyes (CMV.miR204) compared to contralateral eyes injected with the control vector (CMV.EGFP) at PN4 (n=4 animals per stage; Error bars are SEM; n.s.: not significant). Statistical significance (PN30, p-value=0.76; PN60, p-value=0.67) was calculated with a GLM analysis. For each animal, at least 6 retinal sections were analysed and photoreceptor rows were counted in three different parts of each section, distributed within the transduced areas (dorsal eye).

## Figure S3

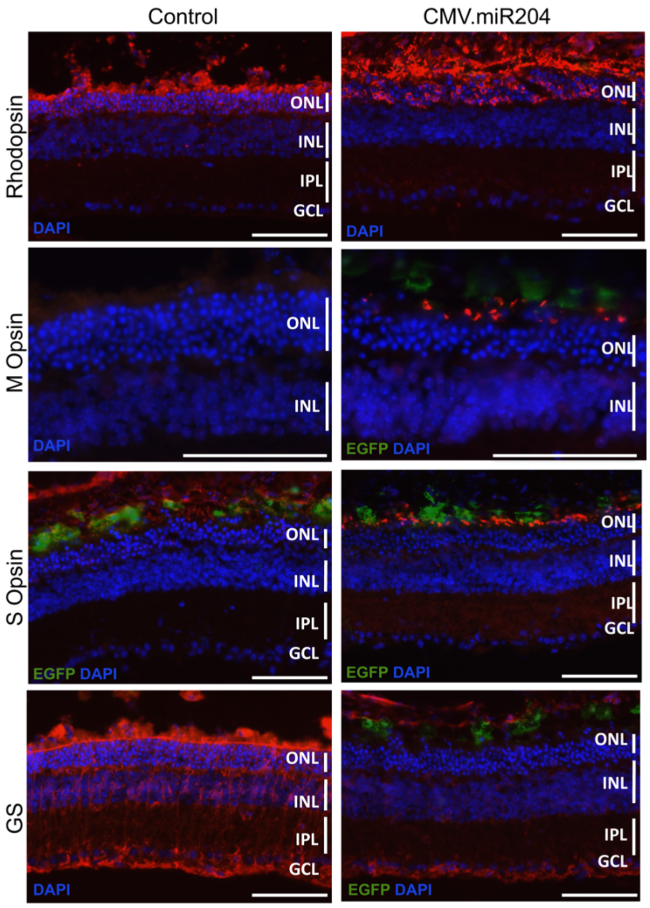


**Figure S3. Expression studies on retinas from 2-month old *RHO*-P347S transgenic mice following AAV-mediated delivery of miR-204 at PN4.**

Immunolabeling for cone photoreceptor markers (M Opsin, S Opsin; in red) and Glutamine Synthetase (GS; in red) on PN60 retinal sections. Nuclei were counterstained with DAPI (blue). Control virus distribution is shown by the EGFP fluorescence (green). For markers with polarised expression (e.g. M Opsin, S Opsin) comparisons were made between corresponding dorsal and ventral areas. Scale bar: 75 µm.

Abbreviations: GCL, ganglion cell layer; INL, inner nuclear layer; ONL, outer nuclear layer; RPE, Retinal Pigment Epithelium.

**Figure S4**

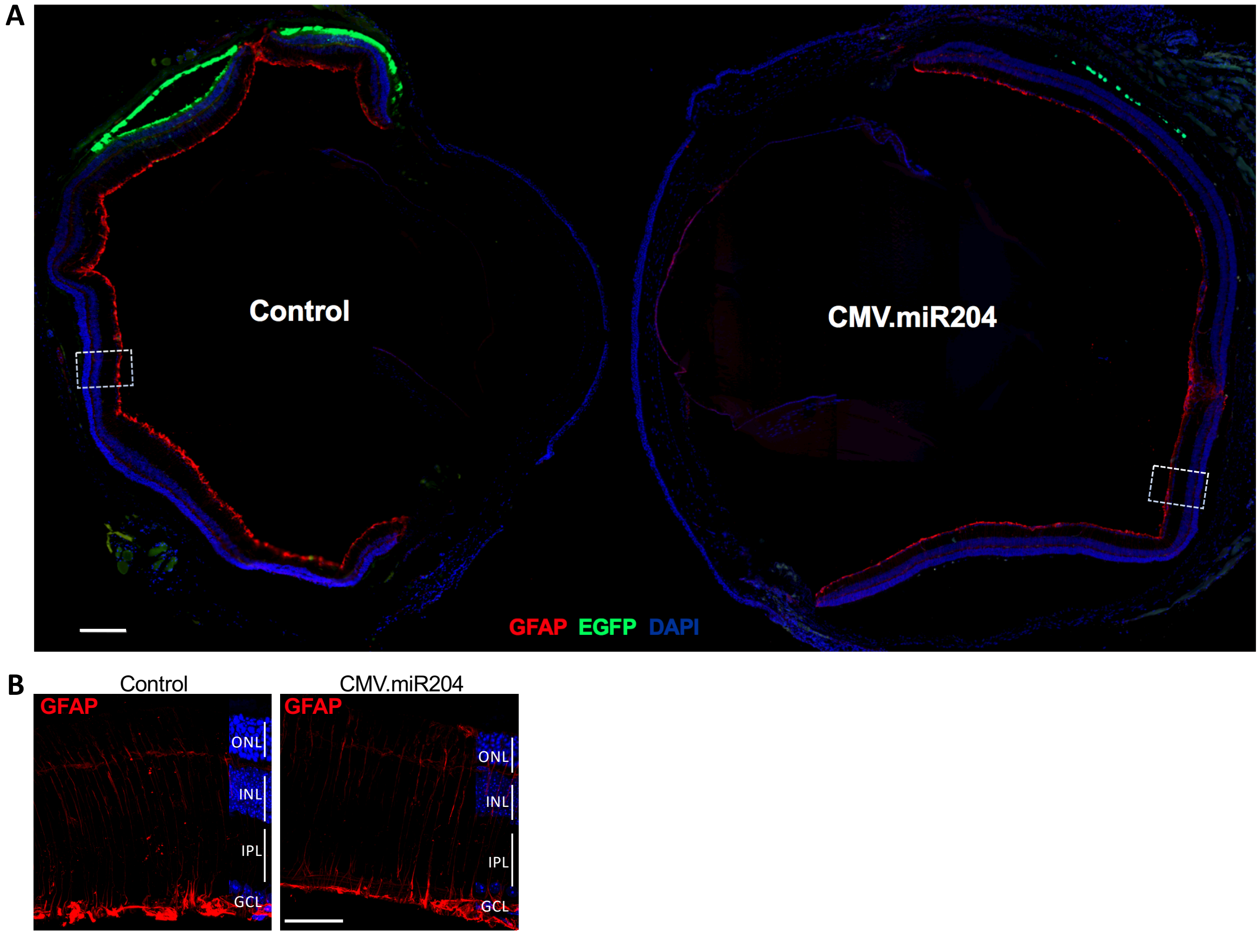


**Figure S4. AAV-mediated delivery of miR-204 at PN4 reduces reactive gliosis in *RHO-P347S* mice.**
**(A)** Panoramic view of entire retina sections at PN30 immunolabelled for Glial Fibrillary Acidic Protein (GFAP; in red). Nuclei were counterstained with DAPI (blue). Control virus delivery is visible by the EGFP fluorescence (green) and is more evident in the RPE of the control-treated eye (left-hand side) injected with the AAV.CMV.EGFP vector ($1.1x10^9$ GC). The contralateral eye was injected with the AAV.CMV.miR204 vector mix ($1x10^9$ GC AAV.CMV.miR204, $1x10^8$ GC AAV.CMV.EGFP). Both sections were present on the same slide and images were acquired as orthogonal projections at the Zeiss Axio Scan.Z1 (20X magnification; Z-stack, 8 slices, 1 $\mu$m intervals). The CMV.miR204-treated eye shows an apparent decrease in retinal gliosis, a physiological response to PR damage, compared to the contralateral control-injected eye. Scale bar: 200 $\mu$m.
**(B)** Confocal microscope images (63X magnification) from the boxed areas in (A). Immunolabeling for GFAP is shown in red. Scale bar: 50 $\mu$m.
Abbreviations: GCL, ganglion cell layer; INL, inner nuclear layer; IPL, inner plexiform layer; ONL, outer nuclear layer.

**Figure S5**

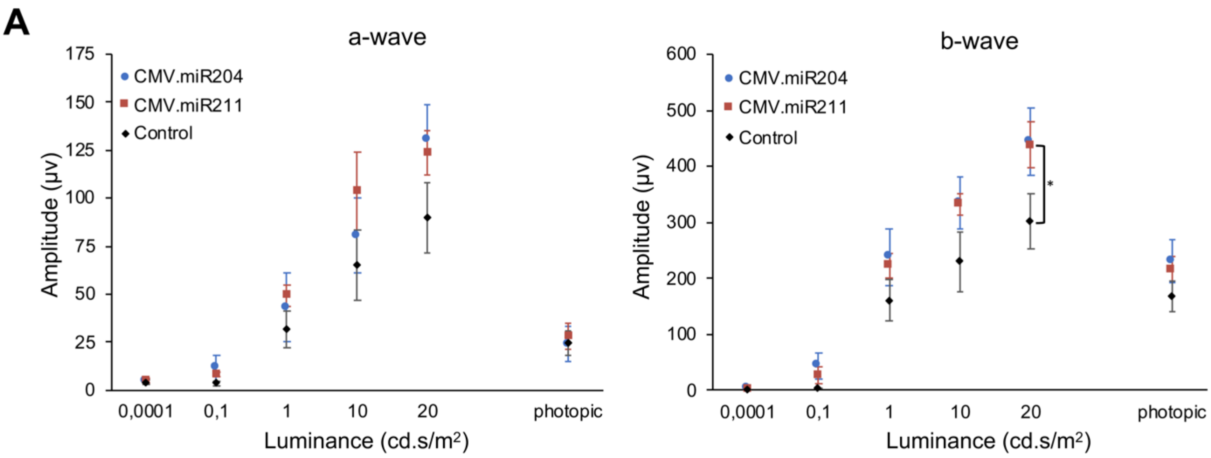


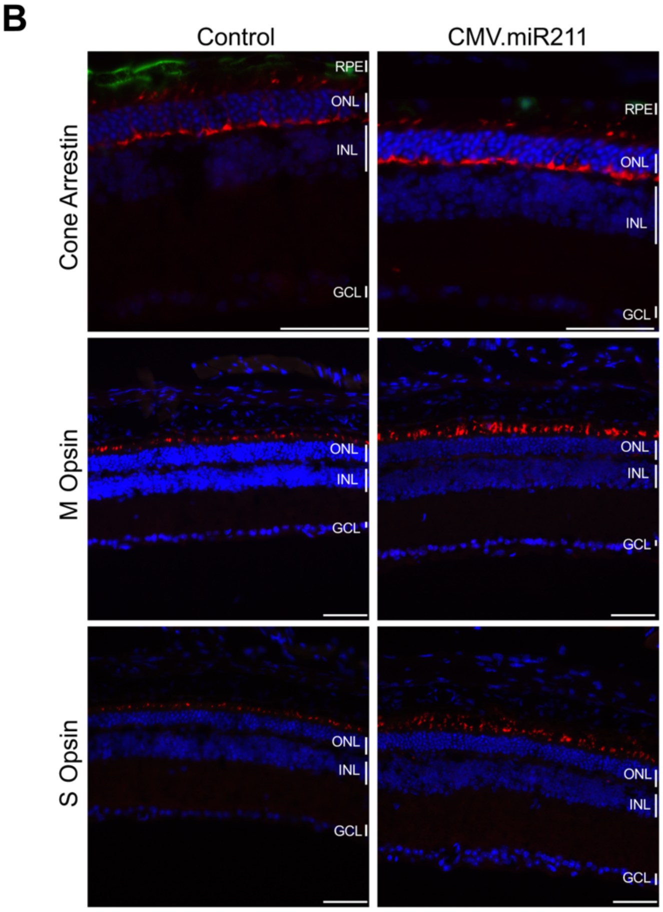


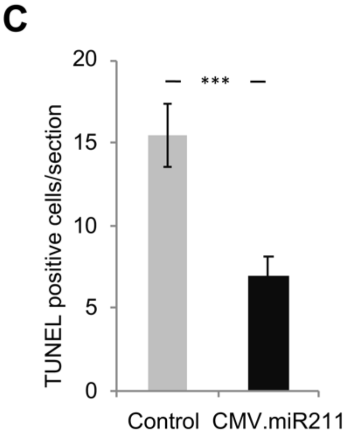


**Figure S5. Functional and molecular analysis of *RHO*-P347S transgenic mice following AAV-mediated delivery of miR-211 at PN4.**

**(A)** A-wave and b-wave ERG responses recorded at PN30 from *RHO*-P347S mice injected with AAV.CMV.miR211, AAV.CMV.miR204 and control vectors at PN4.

**(B)** Immunolabeling for cone photoreceptor markers (M Opsin, S Opsin, Cone Arrestin; in red) on retinal sections from *RHO*-P347S eyes injected with AAV.CMV.miR211. Nuclei were counterstained with DAPI (blue). Control virus distribution (co-injected at a 1:10 ratio with AAV.CMV.miR211) is shown by the EGFP fluorescence (green). Cone opsin expression was better preserved in miR211-treated eyes. Abbreviations: GCL, ganglion cell layer; INL, inner nuclear layer; ONL, outer nuclear layer; RPE, Retinal Pigment Epithelium. Scale bar: 50 μm.

**(C)** TUNEL-positive nuclei at the ONL of eyes injected with the AAV.CMV.miR211 and control vectors. Corresponding sections from serially-sectioned eyes were counted. The mean number of apoptotic photoreceptor cells was significantly reduced in the retina of *RHO*-P347S mice following injection of AAV.CMV.miR211 compared to contralateral control-treated eyes. Error bars are SEM. n=2 retinas. Statistical significance (p-value=0.0001736) was calculated with a GLM analysis.

**Figure S6**

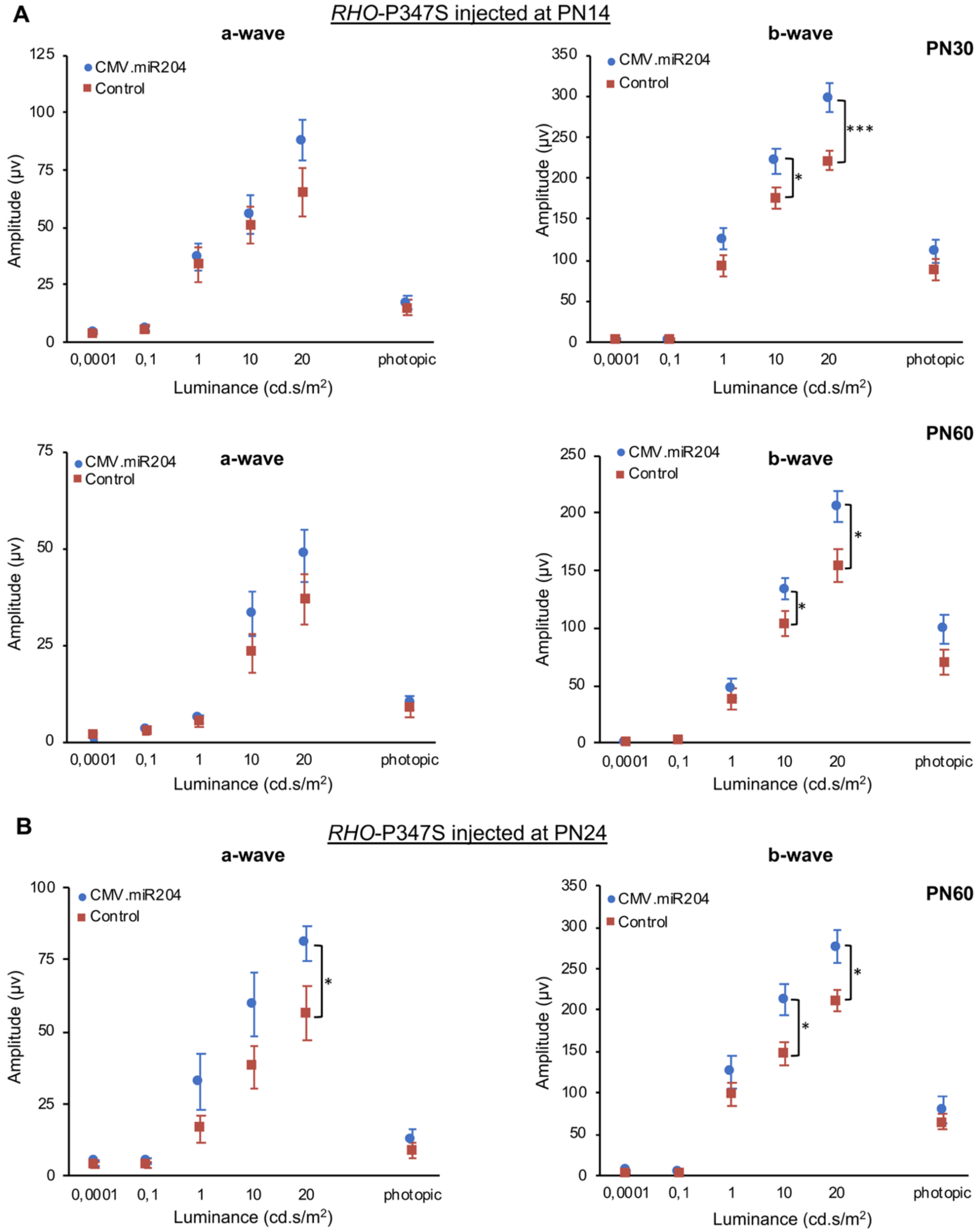


**Figure S6. Protective effect of miR-204 injection at PN14 and PN24 in *RHO*-P347S mice.**

**(A)** ERG responses of *RHO*-P347S mice injected with AAV.CMV.miR204 and the control vector at PN14. ERGs were recorded at PN30 (n=18) and PN60 (n=13). Subretinal injection of AAV.CMV.miR204 at PN14 results in an improvement of retinal function at PN30 and PN60, indicated by a statistically significant increase in b-wave amplitudes vs contralateral eyes injected with the control vector.

**(B)** A- and b-wave ERG responses of *RHO*-P347S mice (n=11) injected with AAV.CMV.miR204 and control vector at PN24. ERGs were recorded at PN60.

Viral doses were: $1x10^9$ GC AAV.CMV.miR204, $1x10^8$ GC AAV.CMV.EGFP per eye; $1x10^9$ GC AAV.CMV.miR204MUT, $1x10^8$ GC AAV.CMV.EGFP per eye. Statistical significance (unpaired Student’s t-test against control vector) is indicated with asterisks (*$p<0.05$, ***$p<0.001$).

**Figure S7**

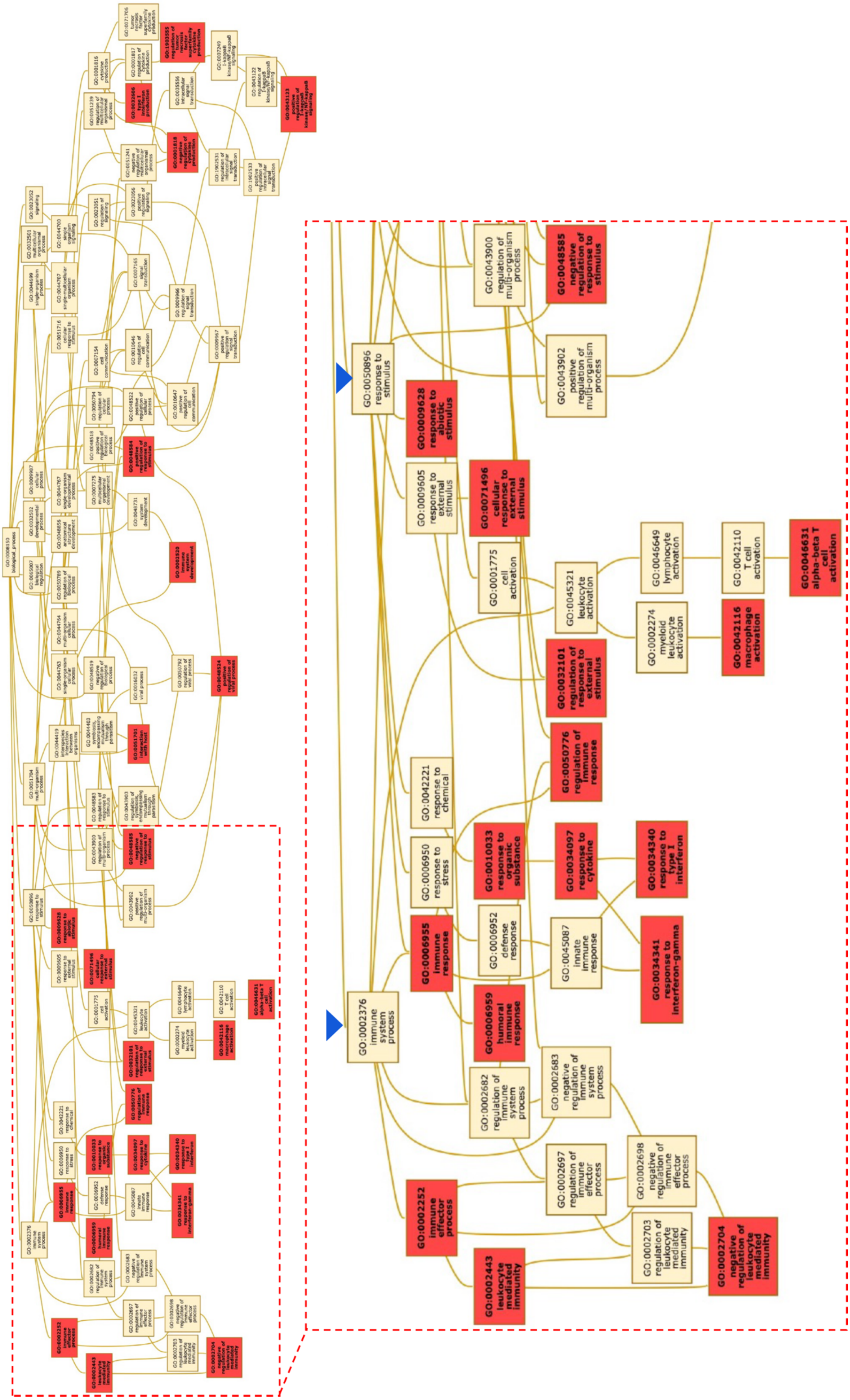


(see legend in the following page)

**Figure S7. GO-view visualisation of BP GO-terms enriched among DEGs downregulated following miR-204 delivery in *RHO*-P347S retinas.**

The graph shows the relationship between the GO terms based on Biological Processes (BPs) and their ancestor terms: 24 clusters (out of 58, red boxes) enriched among the downregulated genes in a statistically significant manner are directly related to the ancestor GO term 'immune system process' (GO:0002376, blue arrowhead). Terms were also related to the 'response to stimulus' (GO:0050896, blue arrowhead) which is a 'parent-term' of the inflammatory response-related terms.

# Figure S8

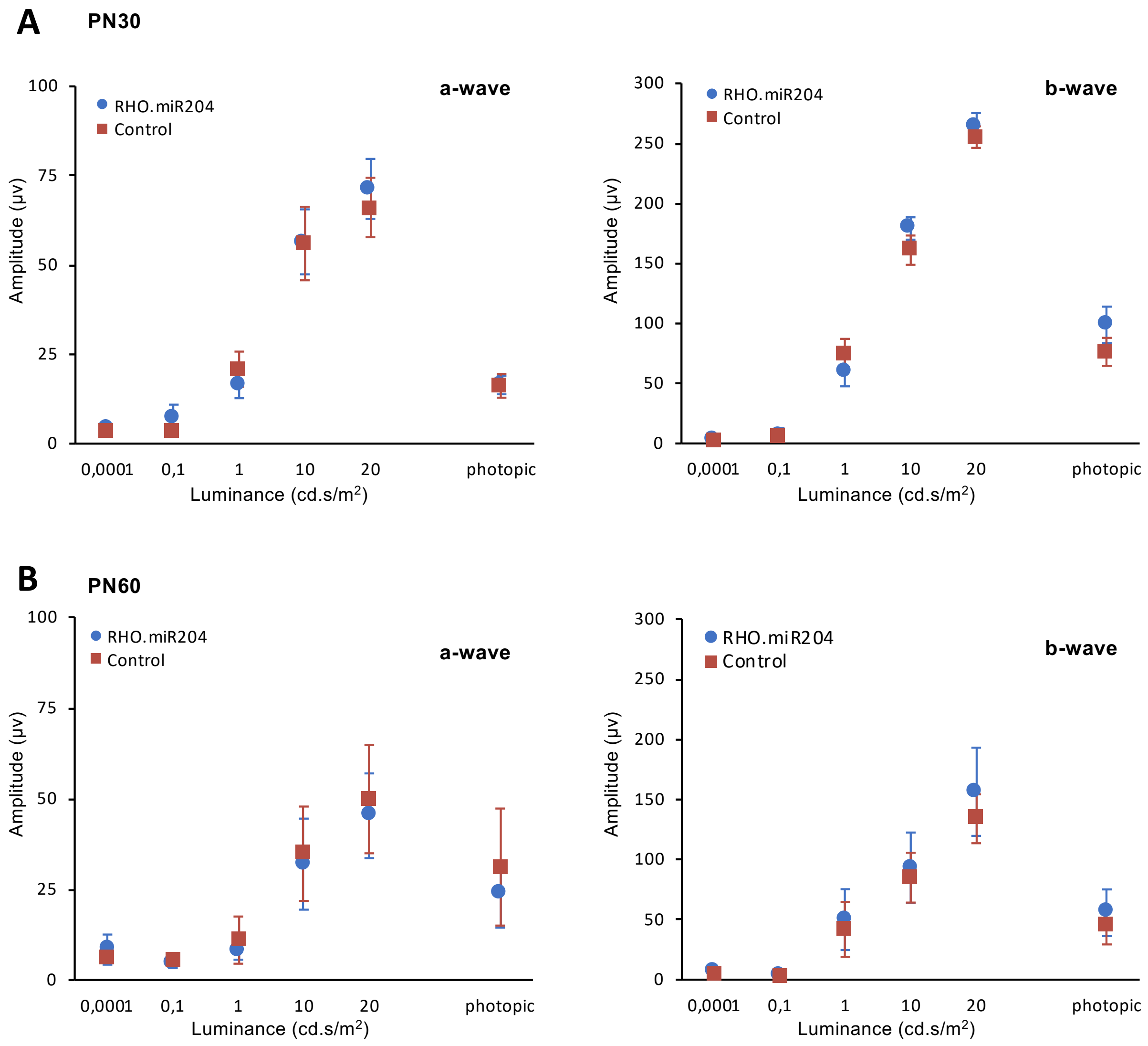


**Figure S8. AAV-mediated delivery of miR-204 using a photoreceptor-specific promoter at PN14 does not improve ERG responses in *RHO*-P347S mice.**

ERG responses of *RHO*-P347S mice injected with the AAV.RHO.miR204 and control vector (AAV.RHO.EGFP) at PN14. ERGs were recorded at PN30 (n=17; panel A) and PN60 (n=5; panel B). No statistically significant improvement of a- and b-wave amplitudes was recorded at PN30 and PN60 in RHO.miR204-treated vs contralateral control eyes. Vector doses were: $1x10^9$ GC AAV.RHO.miR204, $1x10^8$ GC AAV.RHO.EGFP per eye; $1.1x10^9$ GC AAV.RHO.EGFP per control eye.

## Supplemental Tables

(see separate Excel files)

**Table S1. Normalised read counts of the differentially expressed genes between retinas of wild-type c57BL/6 mice injected with the AAV.CMV.miR204 and control AAV.CMV.miR204MUT vector.**
Ensemble ID and Gene Symbol of Differentially Expressed (FDR<0.1) genes between retinas from c57BL/6 mice injected with the AAV.CMV.miR204 (columns c57.Bl6_animalX_OS_CMV.miR204) and AAV.CMV.miR204MUT vector (columns c57.Bl6_animalX_OD_CMV.miR204MUT) in a contralateral manner. Downregulated genes (n=103) are highlighted in green, upregulated genes (n=49) in red. Abbreviations: logFC: log fold change, logCPM: log counts per million, LR: likelihood ratio, p-value: Probability value, FDR: False Discovery Rate.

**Table S2. Gene Ontology enrichment analysis of the differentially expressed genes (DEGs) between retinas of C57BL/6 mice injected with the AAV.CMV.miR204 and control AAV.CMV.miR204MUT vector.**
The Biological Process (BP_FAT) and Cellular Component (CC_FAT) terms enriched among the down-regulated (n=103) and up-regulated (n=49) genes are highlighted in green and red, respectively. For each BP/CC annotation cluster, the GO identifier ("GO:"), associated term ("Term") and Enrichment score are provided. The genes belonging to each term are listed in column D (and their total number in column C under "count"). False Discovery Rate score (FDR) is shown in column E.

**Table S3. Normalised read counts of the differentially expressed genes between retinas of *RHO*-P347S mice (n=3) injected with the AAV.CMV.miR204 and control AAV.CMV.miR204MUT vector.**
Ensemble ID and Gene Symbol of differentially expressed genes (FDR<0.1) between retinas from *RHO*-P347S mice injected with the AAV.CMV.miR204 (columns c57.Bl6_animalX_OS_CMV.miR204) and AAV.CMV.miR204MUT vector (columns c57.Bl6_animalX_OD_CMV.miR204MUT) in a contralateral manner. Downregulated genes (n=76) are highlighted in green, upregulated genes (n=7) in red. Abbreviations: logFC: log fold change, logCPM: log counts per million, LR: likelihood ratio, p-value: Probability value, FDR: False Discovery Rate.

**Table S4. Gene Ontology enrichment analysis of the differentially expressed genes (DEGs) between retinas of *RHO*-P347S mice (n=3) injected with the AAV.CMV.miR204 and control AAV.CMV.miR204MUT vector.**
The Biological Process (BP_FAT) and Cellular Component (CC_FAT) terms enriched among the downregulated (n=76) genes are highlighted in green. For each BP/CC annotation cluster, the GO identifier ("GO:"), associated term ("Term") and Enrichment score are provided. The genes belonging to each term are listed in column D (and their total number in column C under "count"). False Discovery Rate score (FDR) is shown in column E. The small number of up-regulated genes (n=7) precluded a corresponding GO enrichment analysis.

**Table S5. Normalised read counts of the differentially expressed genes between retinas of *RHO*-P347S mice (n=2) injected with the AAV.CMV.miR204 and control AAV.CMV.miR204MUT vector.**
Ensemble ID and Gene Symbol of differentially expressed genes (FDR<0.1) between retinas from *RHO*-P347S mice (animal number 2 and 5) injected with the AAV.CMV.miR204 (columns *RHO*-P347S_animalX_OS_CMV.miR204) and AAV.CMV.miR204MUT vector (columns *RHO*-P347S_animalX_OD_CMV.miR204MUT) in a contralateral manner. Downregulated genes (n=316) are highlighted in green, upregulated genes (n=104) in red.
Abbreviations: logFC: log fold change, logCPM: log counts per million, LR: likelihood ratio, p-value: Probability value, FDR: False Discovery Rate.

**Table S6. Gene Ontology enrichment analysis of the differentially expressed genes (DEGs) between retinas of *RHO*-P347S mice (n=2) injected with the AAV.CMV.miR204 and control AAV.CMV.miR204MUT vector.**
The Biological Process (BP_FAT) and Cellular Component (CC_FAT) terms enriched among DEGs (n=420) are highlighted in green for the downregulated (n=316) and in red for the upregulated genes (n=104). For each BP/CC annotation cluster, the GO identifier ("GO:"), associated term ("Term") and Enrichment score are provided. The genes belonging to each term are listed in column D (and their total number in column C under "count"). False Discovery Rate score (FDR) is shown in column E.

**Table S7. Normalised read counts of the differentially expressed genes (DEGs) between retinas of c57BL/6 (n=3) and *RHO*-P347S mice (n=2) injected with the control vector AAV.CMV.miR204MUT.**
Ensemble ID and Gene Symbol of differentially expressed genes (FDR<0.1) between control-injected retinas from c57BL/6 (columns c57.Bl6_animalX_OD_CMV.miR204MUT) and RHO-P347S mice (columns RHO-P347S _animalX_OD_CMV.miR204MUT). Downregulated genes (n=2158) are highlighted in green, upregulated genes (n=2053) in red. Abbreviations: logFC: log fold change, logCPM: log counts per million, LR: likelihood ratio, p-value: Probability value, FDR: False Discovery Rate.

**Table S8. Gene Ontology enrichment analysis of the differentially expressed genes (DEGs) between retinas of c57BL/6 (n=3) and *RHO*-P347S mice (n=2) injected with the control vector AAV.CMV.miR204MUT.**

The Biological Process (BP_FAT) and Cellular Component (CC_FAT) terms enriched among DEGs (n=4211) are highlighted in green for the downregulated (n=2158) and in red for the upregulated genes (n=2053). For each BP/CC annotation cluster, the GO identifier ("GO:"), associated term ("Term") and Enrichment score are provided. The genes belonging to each term are listed in column D (and their total number in column C under “count”). False Discovery Rate score (FDR) is shown in column E.

**Table S9. List of differentially expressed genes (DEGs; n=290) that show the same trend of down- or upregulation both upon miR204 delivery in *RHO*-P347S mice (compared to delivery of the control vector) and in control-treated wild-type (c57BL/6) retinas (compared to control-treated *RHO*-P347S).**
Genes that are significantly downregulated in both comparisons are highlighted in green. Upregulated genes are highlighted in red. Ensemble ID, Gene Symbol and normalised read counts are shown for DEGs with FDR<0.1. Abbreviations: logFC: log fold change, logCPM: log counts per million, LR: likelihood ratio, p-value: Probability value, FDR: False Discovery Rate.

**Table S10. Gene Ontology enrichment analysis of the genes that are differentially expressed both upon miR204 delivery in *RHO*-P347S mice (compared to delivery of the control vector) and in control-treated wild-type (c57BL/6) retinas (compared to control-treated *RHO*-P347S).**
The Biological Process (BP_FAT) and Cellular Component (CC_FAT) terms enriched among the common DEGs (n=290) are highlighted in green for DEGs downregulated in both comparisons and in red for the commonly upregulated ones. For each BP/CC annotation cluster, the GO identifier ("GO:"), associated term ("Term") and Enrichment score are provided. The genes belonging to each term are listed in column D (and their total number in column C under “count”). False Discovery Rate score (FDR) is shown in column E.
