# Supplementary material for: AAV-miR-204 Protects from Retinal Degeneration by Attenuation of Microglia Activation and Photoreceptor Cell Death
Source: Mol Ther Nucleic Acids. 2019 Nov 18;19:144–56. doi: 10.1016/j.omtn.2019.11.005 (PMC6920266; doi:10.1016/j.omtn.2019.11.005)
Supplement: Document S2. Article plus Supplemental Information [file mmc12.pdf]

Molecular Therapy
Nucleic Acids
Original Article

AMERICAN SOCIETY of GENE & CELL THERAPY

AAV-miR-204 Protects from Retinal Degeneration by Attenuation of Microglia Activation and Photoreceptor Cell Death

Marianthi Karali,[1,2] Irene Guadagnino,[1] Elena Marrocco,[1] Rossella De Cegli,[1] Annamaria Carissimo,[1] Mariateresa Pizzo,[1] Simona Casarosa,[3,4] Ivan Conte,[1] Enrico Maria Surace,[1,5] and Sandro Banfi[1,2]

[1]Telethon Institute of Genetics and Medicine, via Campi Flegrei 34, 80078 Pozzuoli (NA), Italy; [2]Department of Precision Medicine, University of Campania 'Luigi Vanvitelli,' via Luigi De Crecchio 7, 80138 Naples (NA), Italy; [3]Department CIBIO, University of Trento, via Sommarive 9, 38123 Trento, Italy; [4]CNR Neuroscience Institute, via G. Moruzzi 1, 56124 Pisa, Italy; [5]Department of Translational Medicine, 'Federico II' University, via Pansini 5, 80131 Naples, Italy

Inherited retinal diseases (IRDs) represent a frequent cause of genetic blindness. Their high genetic heterogeneity hinders the application of gene-specific therapies to the vast majority of patients. We recently demonstrated that the microRNA miR-204 is essential for retinal function, although the underlying molecular mechanisms remain poorly understood. Here, we investigated the therapeutic potential of miR-204 in IRDs. We subretinally delivered an adeno-associated viral (AAV) vector carrying the miR-204 precursor to two genetically different IRD mouse models. The administration of AAV-miR-204 preserved retinal function in a mouse model for a dominant form of retinitis pigmentosa (*RHO*-P347S). This was associated with a reduction of apoptotic photoreceptor cells and with a better preservation of photoreceptor marker expression. Transcriptome analysis showed that miR-204 shifts expression profiles of transgenic retinas toward those of healthy retinas by the downregulation of microglia activation and photoreceptor cell death. Delivery of miR-204 exerted neuroprotective effects also in a mouse model of Leber congenital amaurosis, due to mutations of the *Aipl1* gene. Our study highlights the mutation-independent therapeutic potential of AAV-miR204 in slowing down retinal degeneration in IRDs and unveils the previously unreported role of this miRNA in attenuating microglia activation and photoreceptor cell death.

INTRODUCTION

Inherited retinal diseases (IRDs), including among others, retinitis pigmentosa (RP), Leber congenital amaurosis (LCA), cone and cone-rod dystrophies, and macular dystrophies, are the most important causes of vision impairment in the working-age population (combined incidence of 1:3,500). These conditions display high genetic heterogeneity, with more than 200 causative genes. Degeneration and death of photoreceptor cells (PRs) are the common landmarks of most IRDs, although the underlying molecular and cellular events are still poorly understood. PR death induces a cascade of reactive processes, such as inflammation and microglia activation, that exacerbate disease progression.

The lack of effective treatments for IRDs remains a major challenge in ophthalmology. Proof-of-concept studies and clinical trials have recently focused on gene-specific therapeutic strategies (GSTSs),[1,2] which are successful in loss-of-function conditions.[3,4] However, the high genetic heterogeneity of IRDs represents a significant limitation in the development and application of GSTSs for a significant fraction of patients. For this reason, mutation-independent therapeutic strategies, acting on common pathways underlying IRDs, are gaining interest as valid alternatives and/or complementary approaches to GSTSs. It is noteworthy that mutation-independent therapeutic strategies could be applicable also to dominant IRDs, for which there is a high, unmet need for therapy compared to recessive conditions. Moreover, mutation-independent strategies can also be used to improve the outcome of gene-specific procedures.

MicroRNAs (miRNAs) are short noncoding RNAs that control fundamental biological processes by targeting networks of functionally correlated genes.[5] In humans, deregulation of miRNA expression correlates with a number of pathological conditions, both multifactorial and monogenic.[6] miRNAs are new targets for therapeutic interventions in a variety of human diseases[7] and are emerging as molecular tools for mutation-independent therapies.

We previously identified miR-204 and its closely related paralog miR-211 among the subset of miRNAs that are significantly expressed in the eye.[8,9] miR-204 is highly abundant in the retina (both in the retinal pigment epithelium [RPE] and neural retina)[8–11] and is also expressed in PRs.[12] We demonstrated that miR-204 is important for proper eye development and function[10,12,13] and can play a pathogenic role in IRDs in humans.[12] The aim of this work was to assess

Received 2 August 2019; accepted 7 November 2019;
https://doi.org/10.1016/j.omtn.2019.11.005.

Correspondence: Sandro Banfi, Telethon Institute of Genetics and Medicine, via Campi Flegrei 34, 80078 Pozzuoli (NA), Italy.
E-mail: banfi@tigem.it
Correspondence: Enrico Maria Surace, Department of Translational Medicine, 'Federico II' University, via Pansini 5, 80131 Naples, Italy.
E-mail: enricomaria.surace@unina.it

This is an open access article under the CC BY license (http://creativecommons.org/licenses/by/4.0/).

the potential of miR-204 to protect PRs from damage caused by different genetic mutations. With the use of an adeno-associated viral (AAV) vector-based strategy, we found that miR-204 administration had a protective effect by attenuating PR death and microglia activation in mouse models of IRDs. We conclude that miR-204 modulation represents a promising strategy for the development of effective and innovative mutation-independent therapies for genetically different forms of IRDs.

RESULTS

Pre-miR-204 Delivered to the Retina via AAV Is Properly Processed and Does Not Cause Alterations of Retinal Function

To deliver miR-204 in mouse retinas, we generated recombinant AAV2/8 vectors expressing the precursor sequence of the murine miR-204 (pre-miR-204) under the control of the constitutive cytomegalovirus (CMV) promoter[14] (AAV.CMV.miR204) (Figure 1A). The AAV2/8 serotype is reported to transduce the RPE and PRs efficiently upon subretinal administration in several species, including mice.[15] Vectors expressing either the miR-204 precursor bearing a mutated seed sequence (AAV.CMV.miR204MUT; Figure S1A) or a reporter (EGFP) gene cassette (AAV.CMV.EGFP) were used as controls (Figures 1A and S1A).

To confirm effective processing in the retina, particularly in PRs, we injected subretinally the AAV.CMV.miR204 vector in eyes of wild-type mice (n = 3) at postnatal day (PN)30. As controls, the contralateral eyes were injected with the AAV.CMV.EGFP. Three weeks later, we observed proper processing of the exogenously provided miR-204 by quantifying the expression level of the mature form of miR-204 in the photoreceptor layers of the transduced area (collected by laser capture microdissection [LCM]) (Figure 1B).

To assess whether miR-204 delivery induces negative effects on healthy retinas, we administered subretinally the AAV.CMV.miR204 vector (mix containing 1×10^9 genome copies [GCs] of AAV.CMV.miR204 and 1×10^8 GC of AAV.CMV.EGFP per eye) in wild-type mice at PN14 (n = 8) and an equal viral load (i.e., 1.1×10^9 GC) of the AAV.CMV.EGFP control in the contralateral eyes (for further details, see Materials and Methods). We tested PR function by full-field electroretinogram (ERG), which did not show significant differences between miR-204- and contralateral control-injected eyes, 1 month postinjection (Figure 1C). Similarly, immunofluorescence (IF) analysis did not reveal significant changes in retinal structure or in the expression of PR markers (Figure 1D). These data indicate that delivery of miR-204 to healthy retinas does not lead to evident signs of retinal dysfunction.

Subretinal Delivery of AAV-miR204 Preserves Retinal Function and Increases Photoreceptor Survival in the *RHO*-P347S Mouse

To test whether miR-204 exerts beneficial effects on IRDs, we used a model for an autosomal-dominant (AD) IRD, namely, the transgenic *RHO*-P347S mouse, which carries a copy of the human *RHODOPSIN(RHO)* gene harboring the proline347-to-serine mutation.[16] An ERG response, albeit severely impaired, can be recorded in *RHO*-P347S up to 2–3 months of postnatal life.[16] We injected *RHO*-P347S mice at PN4 and compared ERG responses at PN30 between miR204-treated eyes (n = 21) and contralateral eyes (n = 22) injected with an equal viral load of control vector (i.e., either AAV.CMV.EGFP or AAV.CMV.miR204MUT) (Figures 2A and 2B). We had previously established that the control vectors (i.e., AAV.CMV.EGFP and AAV.CMV.miR204MUT) elicited comparable ERG responses in *RHO*-P347S mice (Figure S1B). Therefore, we hereafter refer to either of them as “control” for simplicity (vector identities are specified in figure legends). Scotopic a- and b-wave amplitudes at PN30 were preserved in miR204-injected eyes versus controls (Figures 2A and 2B). Photopic ERG responses, recorded in the presence of background light, showed consistently higher amplitudes in treated versus control eyes (Figure 2B). The ERG profiles showed preservation of higher amplitudes compared to controls until PN60, i.e., 2 months postinjection (n = 21) (Figures 2C and 2D).

We did not observe evident differences in the outer nuclear layer (ONL) thickness of the injected eyes by IF assays (Figure S2). However, consistent with photoreceptor function assessments, the expression of PR markers was better preserved in AAV-miR204-treated *RHO*-P347S eyes compared to controls, both at PN30 (Figure 2E) and PN60 (Figure S3). We also observed an apparent reduction of retinal gliosis (Figures S3 and S4) and a statistically significant decrease in the number of apoptotic cells in the ONL of miR204-treated retinas compared to controls (Figure 2F).

We obtained similar beneficial effects also when we injected subretinally miR-211 (1×10^9 GC AAV.CMV.miR211, 1×10^8 GC AAV.CMV.EGFP per eye), a miR-204 paralog, using the same procedure (Figures 1A and S5).

Taken together, these results indicate that subretinal delivery of AAV-miR-204/211 improves retinal function and slows down degeneration in *RHO*-P347S mice.

AAV-miR-204 Delivery Has a Protective Effect Also at More Advanced Stages of Retinal Degeneration

To assess whether miR-204 exerts a protective effect also when delivered in a fully differentiated retina that already displays more advanced PR degeneration, we injected *RHO*-P347S mice at PN14 and PN24 using the above-described experimental approach. Such time points of intervention better represent patient-relevant disease stages. Following injection at PN14, we recorded improved ERG responses at PN30 (n = 18) and PN60 (n = 13) in AAV-miR204-treated eyes versus controls (Figure S6A). Specifically, we recorded an increase in the b-wave amplitude at PN30 that persisted until PN60 (Figure S6A). The a-wave amplitude of the AAV-miR204-injected eyes showed a trend of increase both at PN30 and PN60, albeit without statistical significance (Figure S6A). Similar results were obtained also from *RHO*-P347S animals injected at PN24 (n = 10) and analyzed at PN60 (Figure S6B). These data indicate that miR-204 exerts a protective effect in the *RHO*-P347S mouse model also when administered at more advanced stages of retinal degeneration.

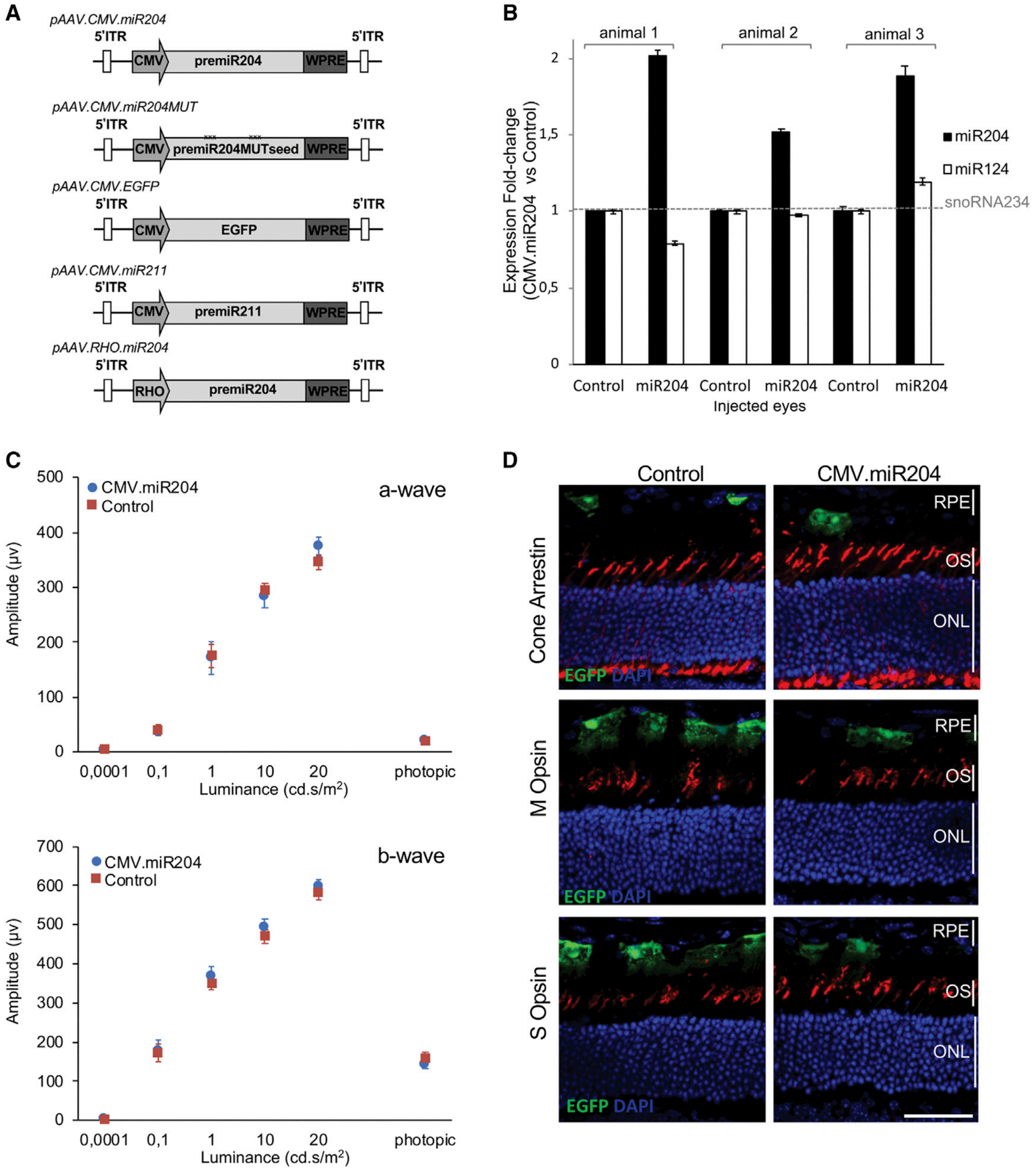

Figure 1. Design of the AAV Vectors, Assessment of Processing, and Effect on Retinal Function of miR-204 Subretinal Injection in Wild-Type Mice

(A) Schematic representation of expression cassettes used for AAV vector production. (B) miRNA expression profile analysis on the LCM-collected photoreceptor layers of three animals injected subretinally with AAV.CMV.miR204 (miR204) and AAV.CMV.EGFP (Control) at PN30 and sacrificed 3 weeks later. Expression levels were determined by TaqMan Real-Time PCR on total RNA and normalized against small nucleolar RNA (snoRNA)234. Levels of miR-124, a retinal-expressed miRNA, were not affected, suggesting that miR-204 administration does not interfere with the global miRNA processing machinery in the retina. Error bars are SEM. (C) ERG responses at PN30 of C57BL/6 mice injected at PN14 with AAV.CMV.miR204 (n = 8) or miR204MUT or AAV.CMV.EGFP (n = 8). No differences were observed. Statistical significance was assesed with an unpaired Student's t test against control vector. (D) Immunolabeling for photoreceptor markers (M Opsin, S Opsin, Cone Arrestin; in red) on retinal sections from wild-type eyes injected with AAV.CMV.miR204. Nuclei were counterstained with DAPI (blue). Control virus distribution (coinjected at a 1:10 ratio with AAV.CMV.miR204) is shown by the EGFP fluorescence (green). Evident differences in retinal structure and expression of PR markers were not observed. ONL, outer nuclear layer; OS, outer segments; RPE, retinal pigment epithelium. Scale bar: 50 μm.

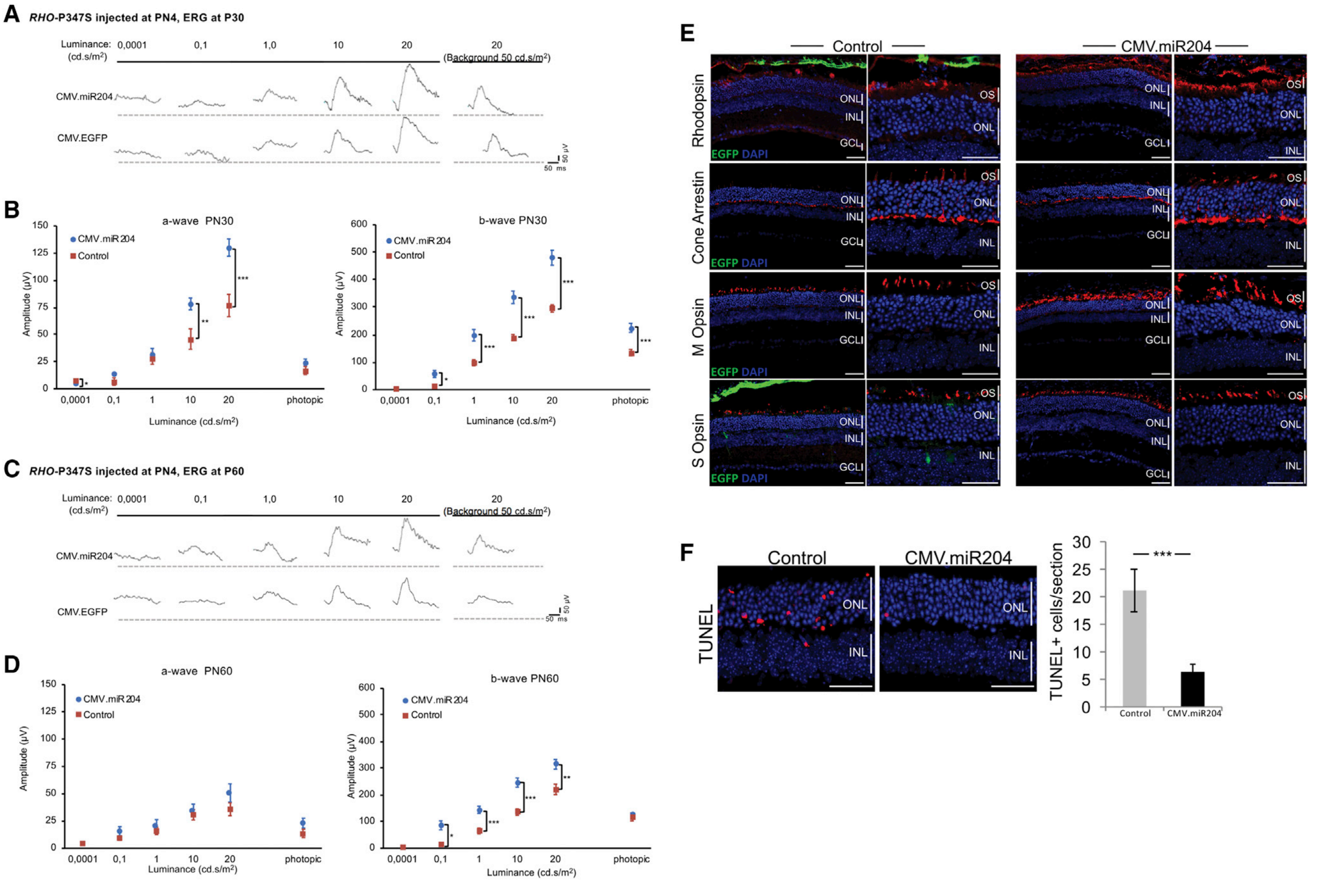

Figure 2. AAV-Mediated Delivery of miR-204 at PN4 Improves ERG Responses and Increases Photoreceptor Survival in *RHO*-P347S Mice

(A and C) ERG traces measured at PN30 (A) and PN60 (C) at increasing luminance (cd·s/m^2) from a representative mouse injected with the AAV.CMV.miR204 vector mix (i.e., 1 × 10^9 GC AAV.CMV.miR204, 1 × 10^8 GC AAV.CMV.EGFP) in one eye and the control vector mix (either 1.1 × 10^9 GC AAV.CMV.EGFP or 1 × 10^9 GC AAV.CMV.miR204MUT, 1 × 10^8 GC AAV.CMV.EGFP) in the contralateral one. (B and D) ERG responses of *RHO*-P347S mice injected with AAV.CMV.miR204 (n = 21) and the control vectors (i.e., AAV.CMV.EGFP or AAV.CMV.miR204MUT, AAV.CMV.EGFP) (n = 22) at PN4. ERGs were recorded at PN30 (B) and PN60 (D). Subretinal injection of AAV.CMV.miR204 results in an improvement of retinal function, which persists up to at least 2 months. Statistical significance (unpaired Student's t test against control vector) is indicated with asterisks (*p < 0.05,**p < 0.01, ***p < 0.001). As a reference, the mean amplitude values of uninjected wild-type animals at different luminance conditions are given. For the a-wave: 0.0001 cd·s/m^2, 6.1 mV; 0.1 cd·s/m^2, 52.8 mV; 1 cd·s/m^2, 213.4 mV; 10 cd·s/m^2, 341.3 mV; 20 cd·s/m^2, 401.1 mV; photopic, 26.4 mV. For the b-wave: 0.0001 cd·s/m^2, 2.3 mV; 0.1 cd·s/m^2, 315.8 mV; 1 cd·s/m^2, 506.3 mV; 10 cd·s/m^2, 732 mV; 20 cd·s/m^2, 876.8 mV; photopic, 188.1 mV. (E) Confocal microscope images from immunolabeling for photoreceptor markers (Rhodopsin, Cone Arrestin, M Opsin, S Opsin; in red) on PN30 retinal sections. Nuclei were counterstained with DAPI (blue). Control virus delivery is visible by the EGFP fluorescence (green) and is more evident in the RPE of the control (AAV.CMV.EGFP)-treated eyes. For markers with polarized expression, comparisons were made between corresponding dorsal and ventral areas. Eyes injected with the CMV.miR204 vector show a stronger staining for PR markers, compared to the contralateral control-treated eyes. (F) Cell death analysis of TUNEL-stained retinal sections from eyes injected with the CMV.miR204 and the CMV.EGFP control vector. Quantification of TUNEL-positive photoreceptor nuclei per section (right) was performed on corresponding sections from serially sectioned, oriented eyes; n = 5 retinas. Error bars are SEM. Statistical significance (GLM analysis) is indicated with asterisks (***p < 0.001). GCL, ganglion cell layer; INL, inner nuclear layer; ONL, outer nuclear layer; OS, outer segments; RPE, retinal pigment epithelium. Scale bars: 50 µm.

Delivery of AAV-miR-204 in the Retina of *RHO*-P347S Counteracts the Activation of Pathways Induced in Retinal Degeneration

To dissect the molecular mechanisms underlying the protective effects of AAV-miR-204 administration to the retina, we carried out a comparative transcriptomic analysis. We injected wild-type (C57BL/6) (n = 3) and *RHO*-P347S (n = 3) mice at PN4 with the AAV.CMV.miR204 vector mix (i.e., 1 × 10^9 GC/eye AAV.CMV.miR204, 1 × 10^8 GC/eye AAV.CMV.EGFP) in one eye and the AAV.CMV.miR204MUT control mix (i.e., 1 × 10^9 GC/eye AAV.CMV.miR204MUT, 1 × 10^8 GC/eye AAV.CMV.EGFP) in the contralateral one. Total RNA was isolated from retinas at PN12, a stage with no detectable signs of PR degeneration in *RHO*-P347S mice.

In wild-type mice, 152 genes were differentially expressed (DE) between the two treatments (103 downregulated, 49 upregulated in miR204- versus control-injected eyes; false discovery rate

[FDR] < 0.1; Table S1). The Gene Ontology (GO) terms enriched among the genes downregulated upon miR-204 delivery were related to neutrophil chemotaxis, regulation of immune response, and cell death, among others (Table S2). On the contrary, there was enrichment for terms associated with visual perception among the upregulated genes (Table S2).

In *RHO*-P347S mice, we found 83 DE genes (DEGs; 76 downregulated, 7 upregulated in miR204- versus control-injected eyes; FDR < 0.1; Table S3) and observed enrichment for terms related to innate immune response among the downregulated ones (Table S4). We observed a very similar enrichment also after removing the RNA sequencing (RNA-seq) data from one animal (mouse number 4), which was highlighted for outlier detection in a principal component analysis (data not shown). A total of 420 genes were DE (FDR < 0.1) when comparing the retinal transcriptome from the two pairs of *RHO*-P347S eyes (Table S5). More than 75% of DEGs (n = 316) were downregulated (Table S5). In GO enrichment analysis (GOEA), with the restriction of the output to biological processes (BP) terms (GOTERM_BP_FAT), we found the most significant enrichment (enrichment score [ES] > 1.5 and FDR < 0.1) for terms related to immune response, innate immune response, and inflammatory responses, among others (Figure 3A; Table S6). Specifically, more than one-half (n = 12) of the top-20 statistically significant enriched BP GO terms were directly related to the parent terms of innate immune and inflammatory response (bold in Figure 3A and Figure S7). Interestingly, also, cell death was among the top-20 enriched BP GO terms (Figure 3A). On the other hand, the 104 upregulated genes (Table S5) were enriched for molecular functions related to the detection of light stimuli and with cellular components related to the photoreceptor outer segment (OS) (Figure 3A; Table S6; ES > 1.5), consistent with the improved ERG responses following AAV-miR-204 administration. Even at this early time point (PN12), *Rho* and S opsin (*Opn1sw*) were among the upregulated genes, in agreement with the preservation of PR morphology and the stronger staining for PR markers observed by IF at PN30 (Figure 2E).

We also compared the expression profiles of control-treated eyes in wild-type (C57BL/6) and dystrophic (*RHO*-P347S) mice. A total of 4,211 genes (2,053 upregulated, 2,158 downregulated) were DE (FDR < 0.1) (Table S7). The DEGs upregulated in the disease model were enriched for BP terms that included innate immune responses, inflammatory processes, and cell death (Table S8; ES > 1.5), suggesting that these pathways are associated with retinal degeneration processes and are induced even at this early preclinical stage in *RHO*-P347S mice. We then determined the overlap between the above-mentioned DEGs (n = 4,211) and those obtained upon miR-204 delivery in *RHO*-P347S (n = 420) (Table S9; Venn diagram in Figure 3B). Sixty-five genes were upregulated, and 225 were downregulated in both conditions (Table S9). Interestingly, the 65 genes that were upregulated both in wild-type mice (compared to the IRD model) and in the AAV-miR204-treated *RHO*-P347S retinas (compared to the control-injected ones) (Table S9; Figure 3B) were significantly enriched for molecular functions related to visual and sensory perception of light stimuli (Table S10). On the other hand, genes that showed reduced relative expression in the above comparisons were enriched for terms related to innate immune response and inflammation (Table S10; Figure 3B).

Taken together, these data indicate that AAV-miR-204 delivery in *RHO*-P347S mice shifts transcriptomic profiles toward those of a healthy retina by downregulating pathways that are induced upon degeneration. More specifically, the pathways of innate immunity, inflammation, and cell death are main effectors through which miR-204 confers neuroprotection in IRDs.

AAV-miR-204 Delivery Dampens Microglia Activation *In Vivo*

The enrichment for innate immunity and inflammation-related pathways among the downregulated genes prompted us to test whether miR-204 modulates microglia activation in the retina. We looked at microglia activation in *RHO*-P347S mice (injected at PN4 with the AAV.CMV.miR204 vector) by double immunostaining for anti-ionized calcium-binding adaptor molecule 1 (Iba1) and either CD68 or major histocompatibility complex class II (MHCII) at PN30 (Figure 4A; data not shown). AAV-miR-204 delivery was associated with a robust reduction in microglia reactivity (Figure 4A). This effect was corroborated both by differences in the morphology of retinal microglia that were more ramified, a conformation characteristic of resting microglia, as well as by a reduced immunostaining for typical markers of phagocytic microglia (e.g., CD68). Consistently, we observed fewer bloated, amoeboid monocytes at the proximity of photoreceptor OS (Figure 4A).

To support a possible direct effect of miR-204 on microglia activation, we demonstrated, by luciferase assay, that miR-204 can bind to *Siglec1*, one of the downregulated miR-204-predicted targets (top 120 DEGs; Table S5; Figure 4C). *Siglec1* encodes for sialoadhesin, a membrane receptor of macrophages and activated monocytes, that was previously shown to promote neuroinflammation in neurodegenerative diseases.[17] Notably, *Siglec1* inactivation in mouse models of neuronal ceroid lipofuscinosis significantly reduced neuron loss and the retinal thinning associated with the condition.[17]

We therefore hypothesize that the protective effect of miR-204 in IRD models is mediated, at least in part, by its impact on microglial activation and on recruited inflammatory macrophages.

miR-204 Contributes to the Control of Photoreceptor Cell Death

As cell death terms were enriched among the DEGs, we looked among the downregulated genes (following miR-204 administration) for direct targets of miR-204 involved in this process. Luciferase assays showed that miR-204 can bind to the 3′ UTR of *Xaf1* (Figure 4C), a miR-204-predicted target among the top-20 DEGs (downregulated) in *RHO*-P347S mice (Table S5). Xaf1 is a proapoptotic factor that negatively regulates XIAP (X-linked inhibitor of apoptosis) activity, thereby sensitizing cells to death triggers.[18] In models of retinal degeneration, the increase of XIAP levels was previously shown to have a protective role.[19,20] Therefore, the direct and early modulation

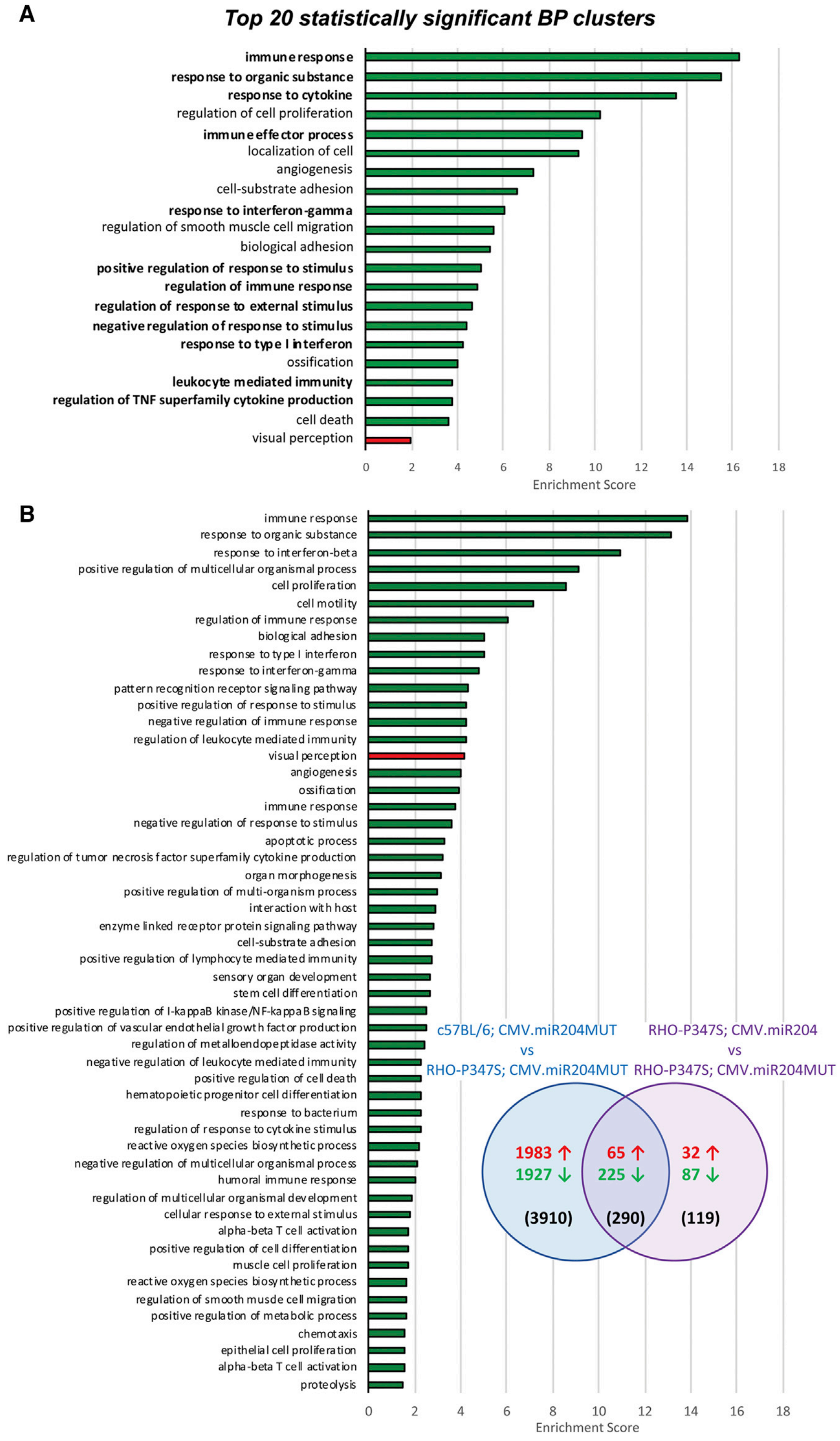

Figure 3. Identification of the Pathways Underlying the miR-204-Protective Effect in *RHO*-P347S Transgenic Mice

(A) Top 20 enriched biological processes (BPs) among the differentially expressed genes in *RHO*-P347S retinas injected with AAV.CMV.miR204. Upregulated genes are significantly enriched for the visual perception BP (red). Downregulated genes are significantly enriched for BPs related to innate immune response, inflammatory response, defense response, and cell death, among others (green). Terms that are directly related to the ancestor terms of "immune system process" (GO: 0002376) and "response to stimulus" (GO: 0050896; ancestor of inflammatory response-related terms) are indicated in bold fonts. Enrichment score values are plotted on the x axis. (B) GOEA of the 290 genes (n = 225 downregulated + 65 upregulated) that are common between the 420 DEGs altered upon AAV-miR-204 expression in *RHO*-P347S (i.e., comparison of the *RHO*-P347S eyes injected with the CMV.miR204 versus miR204MUT control vector; purple circle) and the 4,211 DEGs between control-injected wild-type (C57BL/6) and *RHO*-P347S (blue circle) retinas. The 290 DEGs show the same trend of down- or upregulation in the two comparisons (11 DEGs that show opposite correlation between the two comparisons are not included). The number of DEGs that are either specific of each comparison or overlapping is shown in parentheses. In the Venn diagram, the number of upregulated genes is shown in red and that of downregulated genes in green. All significantly enriched BP terms, in which the common DEGs are involved, are shown (green bars for the downregulated DEGs; red bar for the upregulated DEGs). Terms are ranked according to the enrichment score of each cluster.

of *Xaf1* levels and its downstream impact on anti-apoptotic processes may also account for the observed neuroprotection, consistent with the reduced TUNEL staining observed in treated *RHO*-P347S retinas (Figures 2F and S5C).

Photoreceptor-Specific Delivery of miR-204 in *RHO*-P347S Attenuates Loss of Vision

We sought to assess whether a protective effect of miR-204 delivery could also be obtained using a photoreceptor-specific promoter. To this end, we generated an AAV construct (AAV.RHO.miR204; Figure 1A) in which the precursor sequence of miR-204 is under the control of the human *RHO* promoter[21] that drives transgene expression primarily in rod photoreceptors. A vector (AAV.RHO.EGFP) expressing EGFP under the same promoter was used as control. We injected a group of *RHO*-P347S transgenic mice at PN4 and analyzed retinal function at PN30 (n = 19) and PN60 (n = 11) by ERG (Figure 5). We obtained an improvement of the b-wave ERG response in the RHO.miR204-injected eyes (1×10^9 GC AAV.RHO.miR204 and 1×10^8 GC AAV.RHO.EGFP per eye) compared to the contralateral, RHO.EGFP-injected (1.1×10^9 GC) controls at PN30 (Figure 5A). The increase in the b-wave amplitude persisted until PN60 (Figure 5B); albeit ERG responses of treated eyes

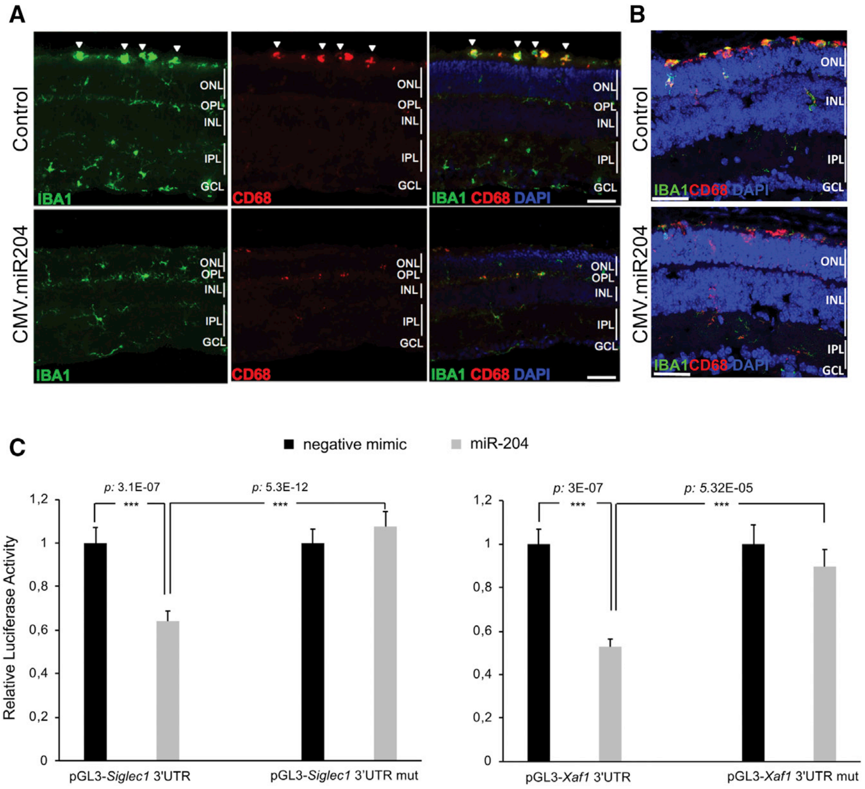

Figure 4. AAV-Mediated Delivery of miR-204 Reduces Phagocytic Microglia Activation in *RHO*-P347S and *Aipl1*$^{-/-}$ Mice

(A) Immunofluorescence staining for microglia markers Iba1 (green) and CD68 (red) on PN30 retinal sections of *RHO*-P347S injected with AAV.CMV.miR204 vector mix (1×10^9 GC AAV.CMV.miR204 and 1×10^8 GC AAV.CMV.EGFP) at PN4. The contralateral eye was injected with the AAV.CMV.EGFP control vector (1.1×10^9 GC). DAPI nuclei counterstaining is shown in blue. Iba1 is a microglia marker, whereas CD68 is a lysosomal marker expressed at high levels in activated/phagocytic microglia. Iba1-positive and CD68-positive, large-bodied ameboid monocytes at the proximity of the photoreceptor outer segments are marked by arrowheads. (B) Microglia marker expression in *Aipl1*$^{-/-}$ animals injected with the AAV.CMV.miR204 vector mix (1×10^9 GC AAV.CMV.miR204, 1×10^8 GC AAV.CMV.EGFP) at PN4 and analyzed at PN12, which corresponds to the early occurrence of photoreceptor death in this model. Microglia reactivity is more prominent in the subretinal space (at the proximity of the photoreceptor OS) in control (CMV.EGFP)-treated eyes. In the miR-204 injected eyes, phagocytic microglia are mainly found at the ONL. (C) Luciferase assays assessing the direct binding of miR-204 to the 3′ UTR of *Siglec1* and *Xaf1*. miR-204 (gray bar) and negative control mimics (black bars) were transfected in HeLa cells together with the luciferase vectors bearing either the wild-type 3′ UTR (pTK-LUC-*X* 3′UTR) or the mutated binding site of the miR-204 seed (pTK-LUC-*X* 3′UTR mut). Relative luciferase activity is reported as fold change to the negative mimic-transfected cells. Data are represented as mean ± SEM. Statistical significance (two-way ANOVA) is indicated with asterisks (***$p < 0.001$; n = 6 observations). GCL, ganglion cell layer; INL, inner nuclear layer; IPL, inner plexiform layer; ONL, outer nuclear layer; OPL, outer plexiform layer. Scale bars: 50 μm.

decreased from 1 to 2 months. a-wave amplitudes also showed a trend of improvement in miR204-treated eyes but without statistical significance.

Administration of AAV.RHO.miR204 delivery at later stages (PN14) did not lead to a statistically significant improvement of a- and b-wave amplitudes at PN30 (n = 17) or at PN60 (n = 5) (Figure S8).

We then sought to determine whether the photoreceptor-specific delivery of miR-204 had an impact on PR viability, PR marker expression, and microglia activation, as observed upon CMV-mediated delivery. By IF analysis and TUNEL assay, we did not detect evident differences in the expression of PR markers (i.e., cone arrestin, Rhodopsin, M opsin, S opsin) and in the extent of cell death between RHO.miR204-injected and control eyes (data not shown). Similarly, we did not observe any significant change in microglia activation following RHO.miR204 delivery (by immunostaining with anti-CD68 and anti-Iba1), thus suggesting that the early effect on immune responses observed in CMV.miR204-treated eyes is likely to be due to the direct effect of miR-204 on innate immune effector cells.

Taken together, these results indicate that the photoreceptor-specific delivery of miR-204 leads to a significant functional recovery in the *RHO*-P347S model, which, however, is lower compared to that achieved by a more widespread retinal delivery of miR-204 using the CMV promoter.

Administration of AAV-miR-204 Increases Photoreceptor Survival in the *Aipl1*$^{-/-}$ Mouse

To assess whether miR-204 impacts on PR degeneration independently of the causative gene, we tested our approach in *Aipl1* homozygous *null* mice.[22] Mutations in the *AIPL1* gene are responsible for a severe form of autosomal recessive IRD (LCA) in humans.[23] In *Aipl1*$^{-/-}$ mice, rod and cone PRs degenerate quickly, leading to a notable reduction of ONL thickness and PR OS[22] and a complete absence of ERG responses at 1 month of age.[22,24] We injected *Aipl1*$^{-/-}$ mice (n = 11) at PN4, using the above-described scheme. We did not record any improvement of retinal function among injected eyes at PN30 (i.e., the earliest time point at which we could perform reliable ERG measurements), most likely due to the severity of degeneration in this animal model (data not shown). This notwithstanding, at PN21, we observed a significant increase in the number of preserved PR rows at the ONL and in the density of PR nuclei in miR204-treated eyes compared to controls (Figure 6A). At this stage, almost invariably, only one row of nuclei is present in the ONL of *Aipl1*$^{-/-}$ eyes injected with the control vector (whereas wild-type retinas have approximately ten rows[25]). We also detected increased staining for rod (Rhodopsin) and cone PR markers in

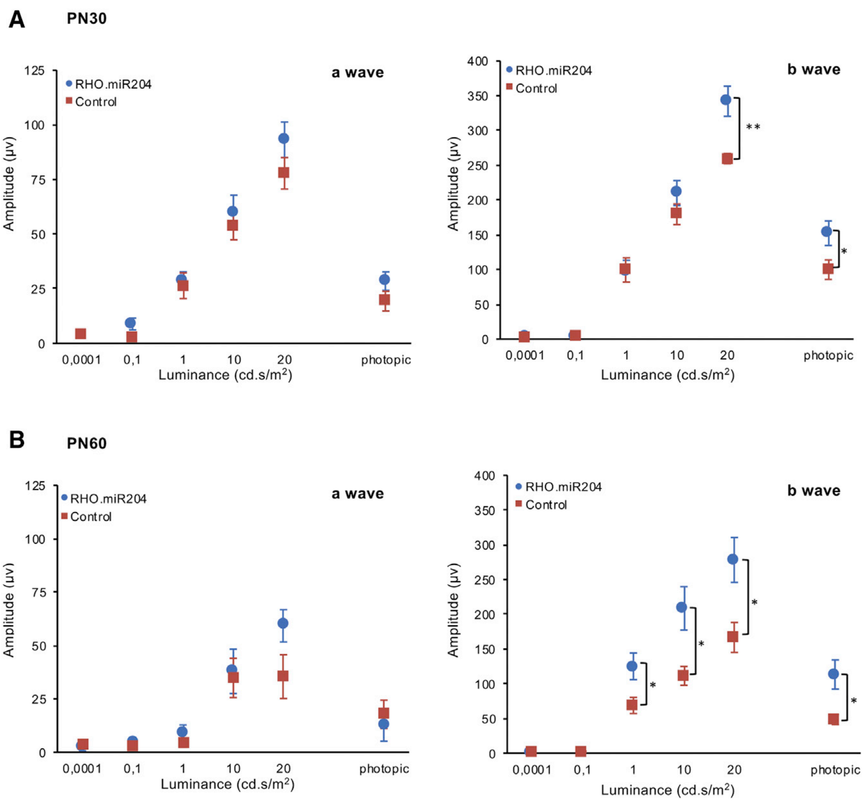

Figure 5. AAV-Mediated Delivery of miR-204 Using a Photoreceptor-Specific Promoter at PN4 Improves ERG Responses

(A and B) ERG responses of *RHO*-P347S mice injected with AAV.RHO.miR204 or AAV.RHO.EGFP at PN4. Mice were recorded at PN30 (A; n = 19) and PN60 (B; n = 11). Subretinal injection of RHO.miR204 results in a statistically significant increase of b-wave amplitudes in miR204-treated eyes compared to control-injected contralateral eyes. Statistical significance (p value), calculated using an unpaired t test against control vector values, is indicated with asterisks (*p < 0.05; **p < 0.01).

miR204-injected eyes (Figure 6B). The structure of cone OS was better preserved in miR204-treated eyes compared to contralateral controls (inset in Figure 6B). Noteworthy, we also observed a reduction of microglia activation in *Aipl1*$^{-/-}$ miR204-treated eyes (Figure 4B), indicating that its modulation by miR-204 is independent of the genetic mutation.

Altogether, these data show that miR-204 exerts a mutation-independent protective effect in IRD models by acting on multiple pathways.

DISCUSSION

This study demonstrates that delivery of miR-204 in the subretinal space attenuates photoreceptor degeneration in two genetically different IRD mouse models. miR-204 exerts its neuroprotective effect by acting on common pathways to which IRDs converge, irrespective of the initial disease-causing mutation.

A single subretinal injection of an AAV vector expressing miR-204 preserves PR marker expression and viability, ultimately recovering visual function in *RHO*-P347S mice (Figure 2). The improved ERG responses are mainly attributable to a better preserved PR function and to an attenuation of the degeneration, even though the reduction of PR demise did not elicit a visible increase in ONL thickness at the analyzed stages (Figure S2). The severity and fast progression of degeneration of the models employed in this study (*RHO*-P347S and *Aipl1*$^{-/-}$) are appropriate to demonstrate effects of novel treatments but are not ideal for long-term assessments of the effect of miR-204 delivery on the ONL morphology. Alternative animal models (even from other organisms) that allow extended follow-up periods (i.e., with a slower progression of degeneration, ideally combined with a larger lifespan) will be necessary to address fully the discrepancy between the preservation of visual function and the apparent lack of morphological protection observed after miR-204 delivery.

Protective effects were also obtained by interventions at later time points (i.e., at PN14 or PN24), when fully differentiated retinas exhibit signs of PR degeneration (Figure S6). These injections resulted in a reduced improvement of visual function, as a higher number of photoreceptors are irreversibly damaged or lost by then. Nevertheless, the functional recovery by injections after disease onset is relevant, as it supports the clinical potential of the approach at patient-relevant stages of disease progression.

Our results indicate that miR-204 delivery to IRD mouse models acts on multiple correlated processes associated with innate immunity, inflammatory responses, and cell death. We believe that miR-204 has a primary effect on these pathways, because they were significantly modulated already before the onset of degeneration and were also impacted in wild-type retinas. The latter observation may be, in part, due to the effects of miR-204 in alleviating damage associated with subretinal injection. Based on the gathered experimental evidence, we propose that miR-204 exerts its neuroprotective effect in IRDs through at least two mechanisms.

First, miR-204 slows down degeneration by acting directly on PR death. In addition to the functional (Figures 2A and 2D) and histological evidence (Figures 2E and S3), this mechanism is corroborated by the reduced TUNEL staining in retinas of miR204-treated *RHO*-P347S mice (Figure 2F). We also demonstrated that miR-204 can bind the 3′ UTR of *Xaf1* (Figure 4C). Xaf1 is a proapoptotic protein that promotes cell death by negatively regulating XIAP,[18] a potent inhibitor of apoptosis.[26] It is therefore plausible that miR-204 protects

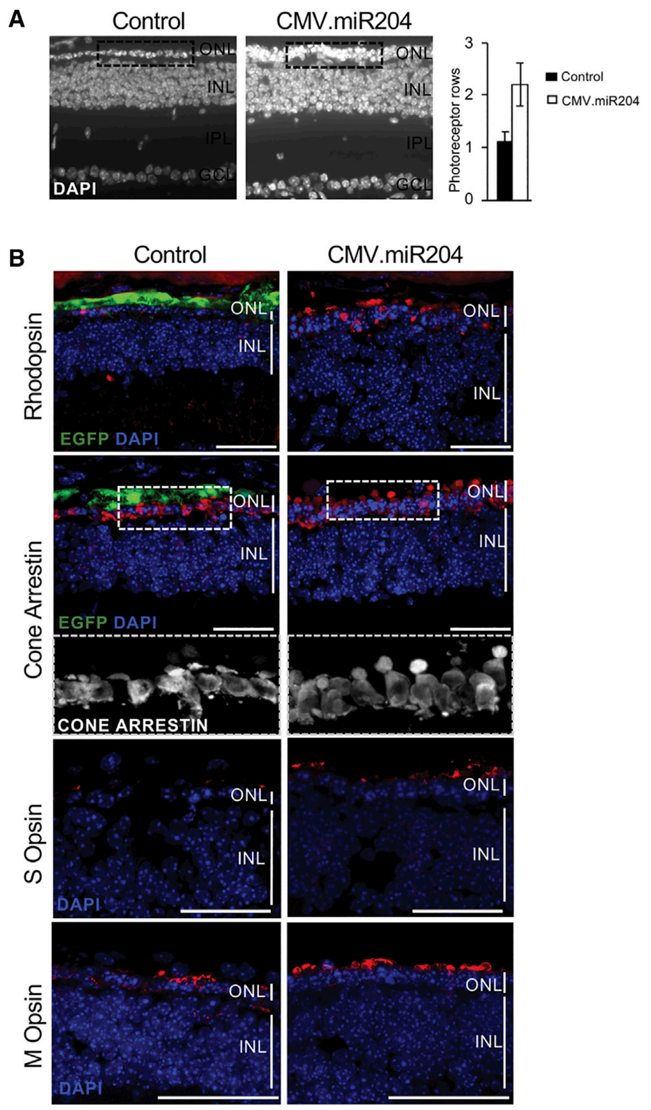

Figure 6. AAV-Delivered miR-204 at PN4 Increases Photoreceptor Survival in the *Aipl1*$^{-/-}$ Mouse

(A) Nuclei staining (DAPI) of *Aipl1*$^{-/-}$ retinal sections at PN21 following subretinal delivery of miR-204 at PN4. Almost invariably, only one row of nuclei is present in the ONL of *Aipl1*$^{-/-}$ eyes injected with the control CMV.EGFP vector (left dashed box inset). There is an increase in the number of rows and density of nuclei at the ONL of the contralateral eye injected with the AAV.CMV.miR204 vector mix (right dashed box inset). The other retinal layers (INL, IPL, and GCL) appear to be unaffected. Plot shows the average number of photoreceptor nuclei rows in miR204-treated eyes compared to contralateral eyes injected with the control vector (n = 11; error bars are SEM; p = 0.06). (B) Confocal microscopy images of immunolabeling for rod (Rhodopsin; in red) and cone photoreceptor markers (Cone Arrestin, M Opsin, S Opsin; in red) on retinal sections at PN21. Control virus distribution is shown by the EGFP fluorescence (green). DAPI nuclei counterstaining is shown in blue. An increased staining for photoreceptor markers is observed at the ONL of eyes injected with the AAV.CMV.miR204 vector mix compared to contralateral control (CMV.EGFP)-treated eyes. Dashed box insets: close-up of the staining for cone arrestin in the framed area. Viral doses were the following: 1×10^9 GC AAV.CMV.miR204/1×10^8 GC AAV.CMV.EGFP per eye and 1.1×10^9 GC AAV.CMV.EGFP per eye). GCL, ganglion cell layer; INL, inner nuclear layer; IPL, inner plexiform layer; ONL, outer nuclear layer. Scale bars: 50 μm.

PRs from cell death, in part, by downregulating *Xaf1* expression. Given that XIAP also limits inflammasome activation,[27] it is reasonable to propose that downregulation of *Xaf1* enhances the XIAP-mediated inhibition of inflammatory responses. In support of the beneficial role of XIAP, its overexpression in the retina conferred protection in models of degeneration[19,20] and enhanced survival of transplanted photoreceptors in degenerating retinas.[28]

Second, miR-204 attenuates disease progression by dampening microglia activation in response to PR dysfunction and death (Figures 3 and 4). Administration of miR-204 in *RHO*-P347S mice strongly inhibits the cascade of innate immune responses and inflammation mounted at the onset of degeneration (Figure 3A). It is noteworthy that downregulation of the above-mentioned biological pathways occurs already at PN12, an early, "preclinical" stage of degeneration in *RHO*-P347S mice. Microglia reactivity is induced by the innate immune system in response to PR death and contributes primarily to the clearance of dysfunctional and dying photoreceptors. However, as shown in other neurodegenerative contexts,[29] prolonged or excessive microglial activation instigates inflammatory responses and exacerbates degeneration by the constant secretion of proinflammatory compounds and by the phagocytosis of nonapoptotic, living PRs.[30,31] In agreement with their neurotoxic potential, several studies reported that modulation of microglia activation and innate immune response holds promise for the treatment of retinal degeneration.[32] Approaches to limit microglia over-reactivity should not, however, interfere with the homeostatic function of retinal immune effectors. Based on our results, we propose that miR-204 acts as a modulator of innate immune responses and of proinflammatory pathways that exacerbate disease progression. As miRNAs fine tune functionally correlated pathways,[5] the sustained retinal transduction provided by AAV-miR-204 delivery may represent an alternative to more transient and drastic pharmacological approaches in suppressing microglia reactivity without compromising tissue homeostasis.

The data obtained with the use of the *RHO* promoter demonstrate that the neuroprotective effect of miR-204 in IRD models is exerted, at least in part, through a PR-autonomous mechanism. The administration of miR-204 under a ubiquitous promoter had a stronger effect, presumably through the pleiotropic action of this miRNA on multiple cell targets (e.g., RPE, photoreceptors, microglia). Therefore, the dampening of microglial reactivity observed upon AAV.CMV.miR204 delivery is most likely due to a direct role of this miRNA on microglia activation rather than secondary to events occurring in PRs (e.g., a reduced recruitment of microglia due to a decrease in "eat me" signals produced by PRs). In view of translational applications in additional (pre)clinical models, the risk-benefit balance between a cell-targeted approach and the use of a ubiquitous promoter would need to be carefully assessed.

The described delivery route (i.e., subretinal injection), vehicle (i.e., AAV vectors), and disease stage at intervention (i.e., PN24, advanced postnatal stages) are compatible with the development of a clinical protocol for human translational purposes (e.g., subretinal delivery in patients).[33] Further enhancements could derive by the testing of different miR-204 molecules (e.g., miRNA mimics) and conditions (e.g., higher vector dose, inoculations in multiple injection spots) and their combinations in preclinical models of IRDs. With the consideration of the effect of miR-204 on multiple disease mechanisms common to genetically different IRDs, this miRNA can represent a mutation-independent therapeutic agent that dampens disease-amplifying processes, thus also supporting gene-specific replacement approaches. This is particularly relevant for dominant conditions that cannot be treated by gene replacement due to the gain-of-function/dominant-negative effects of the mutated allele. Multifactorial forms of retinopathies with an established innate immunity etiology (such as age-related macular degeneration [AMD] or diabetic retinopathy) may also benefit from the proposed strategy. Future research should experimentally address this perspective.

MATERIALS AND METHODS

Plasmid Construction

Recombinant AAV vectors containing the murine precursor sequence of miR-204 (or miR-211) under the CMV promoter were constructed by a two-step cloning protocol. Initially, the cassette containing the precursor of miR-204 (or miR-211) was amplified from mouse genomic DNA using the following set of oligonucleotides: 5′-ATAAGAATGCGGCCGCCTGTTCAGGACTTGGCTAAG-3′ and 5′-CGCGGATCCAACATGGGGTTGTTAATCTG-3′ for miR-204; 5′-ATAAGAATGCGGCCGCTCTGACCATGCAATCACAG-3′ and 5′-CGCGGATCCAATGGATCAGGGTGGCATC-3′ for miR-211. The obtained amplimers were subcloned in the TOPO TA Cloning Vector (Invitrogen) and released following digestion with Not I and BamH I. The fragment was then cloned into the Not I–BamH I sites of the pAAV2.1-CMV-EGFP plasmid[15] and used for the production of AAV2/8 vectors.

The control vector bearing the precursor of miR-204 with a mutated seed sequence was generated by purine-pyrimidine changes in both arms of the precursor to maintain the same complementarity and hairpin structure (Figure S1A). This was achieved with two sequential steps of site-directed mutagenesis using primer extension. The first reaction introduced the modified seed sequence of the mature miRNA (5′-GGAAAGGG-3′ instead of 5′-TTCCCTTT-3′ at chr19: 22,750,610–22,750,617) and the second introduced a sequence complementary to the newly mutated seed at the 3p arm of the precursor hairpin (5′-CCCTTTCA-3′ instead of 5′-AAAGGGAC-3′ at chr19: 22,750,660–22,750,667) (Figure S1A). The internal primer pairs containing the desired mutation and complementary ends were 5′-CGTGGACGGAAAGGGGTCATCCTATGCCTG-3′, 5′-GGATGACCCCTTTCCGTCCACGAGTCACATG-3′ and 5′-GGAAGGCCCCTTTCAGTTCAATTGTCATCAC-3′, 5′-ATTGAACTGAAAGGGGCCTTCCCAGCCTCC-3′. The external flanking primers, used in combination with the internal mutagenic primers, were the ones described above for the amplification of the miR-204 precursor.

The vector bearing the precursor miR-204 under the control of a photoreceptor-specific promoter (pAAV.RHO.miR204) was generated by exchanging the CMV promoter of pAAV.CMV.miR204 with the human *RHO* promoter sequence.[21] Briefly, the sequence corresponding to the human *RHO* promoter was released from the pAAV2.1-*RHO*-EGFP plasmid[15] by restriction with Nhe I and Not I and was cloned in the pAAV.CMV.miR204 backbone, previously digested with the same enzymes.

AAV Virus Production

Recombinant AAV2/8 viruses were produced by the TIGEM Vector Core as reported.[34] Physical titers (GCs per milliliter [GC/mL]) of each viral preparation were determined by TaqMan PCR quantification.

Animal Models and Procedures

For subretinal injections in the *RHO*-P347S background, pups were obtained by crossing the *RHO*-P347S transgenic[16] with C57BL/6 mice. For injections in the $Aipl1^{-/-}$ background, pups were obtained by crosses between homozygous $Aipl1^{-/-}$ mice.[22] The C57BL/6 strain was used as a wild-type control line.

Studies were conducted in accordance with the institutional guidelines for animal research and approved by the Italian Ministry of Health (D.L. 116/92 art. 7; protocol number: 650/2018-PR). Mice were maintained under specific pathogen-free (SPF)-like conditions at the TIGEM Animal Facility. Surgical procedures were performed under anesthesia, and all efforts were made to minimize suffering. Viral vectors were delivered subretinally in the dorsal retinal areas via a trans-scleral transchoroidal approach.[35] Eyes were injected with 1 μL of an AAV vector/vector mix (specified in each figure) containing a total of 1.1×10^9 viral GCs.

Electroretinography

Electrophysiological recordings were performed as previously described.[36] Mice were dark adapted for 3 h and accommodated in a stereotaxic apparatus under dim red light. Pupils were dilated with a drop of 1% tropicamide (Alcon Laboratories, Fort Worth, TX, USA), and the body temperature was maintained at 37.5°C. ERGs were evoked by 10-ms flashes of different light intensities ranging from 10^{-4} to 20 $cd \cdot s/m^2$, generated through a Ganzfeld stimulator (CSO, Florence, Italy). To minimize the noise, three different responses evoked by light were averaged for each luminance step (the time interval between light stimuli was 4–5 min). Electrophysiological signals were recorded with gold-plated electrodes inserted under the lower eyelids in contact with the cornea. Electrodes in each eye were referred to a needle electrode inserted subcutaneously at the level of the corresponding frontal region. The different electrodes were connected to a two-channel amplifier. Amplitudes of a- and b-waves were plotted as a function of increasing light intensities. After completion of responses obtained in dark-adapted conditions

(scotopic), the recording session proceeded to dissect the cone pathway mediating the light response (photopic). To this end, ERG responses to light of 20 cd·s/m^2 were recorded in the presence of a continuous background light (set at 50 cd·s/m^2). For each group, the mean a- and b-wave amplitude was plotted as a function of luminance (transfer curve) under scotopic and photopic conditions.

Immunostaining

Immunostaining for retinal and microglial markers was performed as reported.[37] Frozen retinal sections were washed once with PBS and then fixed for 10 min in 4% paraformaldehyde (PFA). Sections were then permeabilized either for 15 min in PBS containing 1% IGEPAL® CA-630 (Sigma-Aldrich, St. Louis, MO, USA; for anti-Rhodopsin, anti-cone arrestin, anti-glutamine synthetase) or in citrate buffer (for the anti-M and -S opsin). Blocking solution containing 10% normal goat serum (Sigma-Aldrich, St. Louis, MO, USA) was applied for 1 h. Primary antibodies were incubated overnight at 4°C. Secondary antibodies (Alexa Fluor 594, anti-rabbit or anti-mouse, 1:1,000; Molecular 20 Probes, Invitrogen, Carlsbad, CA, USA) were applied for 1 h. Permeabilization and blocking of sections for anti-Iba1, anti-CD68, anti-MHCII, and anti-glial fibrillary acidic protein (GFAP) staining were performed for 1 h in 0.3% Triton/4% normal goat serum. Antibodies were then incubated overnight at 4°C in 0.1% Triton/2% normal goat serum (for dilutions and providers, see below). DAPI (Vectashield; Vector Labs, Peterborough, UK) was used for nuclei counterstaining. Sections were photographed using Zeiss (LSM 710) confocal microscopy or a Zeiss Axiocam (Carl Zeiss, Oberkochen, Germany). Panoramic images of entire retinal sections were acquired as orthogonal projections at the Zeiss Axio Scan.Z1.

The primary antibodies used were anti-human cone arrestin (hCAR; 1:10,000; AB15282; Millipore, Burlington, MA, USA), anti-Opn1mw (anti-M opsin; 1:200; AB5405; Millipore, Burlington, MA, USA), anti-Opn1sw (anti-S opsin; 1:200; AB5407; Millipore, Burlington, MA, USA), anti-Rhodopsin (1:5,000; Abcam, Cambridge, UK), anti-GFAP (1:400; Z0334; Dako Agilent, Santa Clara, CA, USA), anti-glutamine synthetase (anti-GS; 1:500; MAB302; Millipore, Burlington, MA, USA), anti-Iba1 (1:300; 019-19741; Fujifilm Wako Pure Chemical, Osaka, Japan), anti-MHCII (1:200; MCA46GA; Bio-Rad, Hercules, CA, USA), and anti-CD68 (1:500; MCA1957T; Bio-Rad, Hercules, CA, USA).

Histological Analysis

Enucleated eyeballs were fixed overnight by immersion in 4% PFA. Prior to enucleation, cautery of the sclerae was used to orient the eyes, with respect to the injection site, at inclusion. Eyes were infiltrated with 30% sucrose for cryopreservation and embedded in tissue-freezing medium (O.C.T Matrix; Kaltek, Padua, Italy) in pairs (i.e., left and right eye) to facilitate comparative analysis. For each eye, 10 μm-thick serial sections were cut along the horizontal plane. Sections were progressively distributed on 10 slides (each slide contained sections representative of the whole eye at different levels) and stained with DAPI for nuclei visualization (Vectashield; Vector Labs, Peterborough, UK).

Cell Death Assay (TUNEL)

Apoptotic nuclei in frozen retinal sections were detected by the TUNEL kit, according to the manufacturer's instructions (*In Situ* Cell Death Detection Kit, TMR red; Roche-Merck KGaA, Darmstadt, Germany).

RNA Extraction, Library Preparation, Deep Sequencing, and Computational Analysis

Total RNA was extracted from mouse retinas using the miRNeasy kit (QIAGEN, Hilden, Germany). RNA-seq library preparation and sequencing were performed as previously described.[38] The RNA-seq data are deposited in NCBI's Gene Expression Omnibus (GEO: GSE121785). Data analysis was performed as reported.[38] The threshold for statistical significance was FDR <0.1.

GOEA

GOEA[39] was performed on the list of upregulated and downregulated genes, separately. The Database for Annotation, Visualization and Integrated Discovery (DAVID) online tool (v. 6.7) was used by restricting the output to BP (BP_FAT) and Cellular Component (CC_FAT) terms, with a significance threshold of FDR <10% and ES >1.5. Redundant terms and commonly encountered categories were eliminated. The visualization of GO-term interconnections was performed using GOView (http://www.webgestalt.org/GOView/).

Target Prediction and Luciferase Assays

Sequence-based miRNA target-prediction databases (e.g., microT-CDS,[40] http://diana.imis.athena-innovation.gr/DianaTools/index.php?r=microT_CDS/index; TargetScan,[41] http://www.targetscan.org/) and coexpression-based tools for systematic miRNA target recognition (e.g., Co-expression Meta-analysis of miRNA Targets [CoMeTa];[42] http://cometa.tigem.it/) were used to annotate miR-204-predicted targets.

Luciferase assays were performed as described.[9] To test the direct regulation of the 3′ UTR of target genes by miR-204, the 3′ UTR-bearing vector was cotransfected with a synthetic miR-204 mimic (Dharmacon, Lafayette, CO, USA). Appropriate control vectors, in which the sequence of the candidate miRNA binding site at the 3′ UTR (i.e., predicted to be recognized by the miR-204 seed region) was mutagenized to abolish binding, were used in parallel.

Statistical Analysis

For ERG experiments, unpaired Student's t test was performed against control-treated eyes. For experiments, a one-way ANOVA, followed by Tukey *post hoc* procedures, was used for comparisons. For luciferase experiments, a two-way ANOVA and Tukey *post hoc* procedures were used to compare groups. For discrete data, the likelihood ratio test for general linear model (GLM) was performed. All values are expressed as means ± SEM.

LCM

Frozen retinal sections on polyethylene naphthalate (PEN)-membrane slices were microdissected using a Laser MicroDissection

(LMD) 6500 microscope. Slices were fixed in cold 75% ethanol (EtOH) in diethyl pyrocarbonate (DEPC)-treated water (2 min), washed twice in DEPC-treated water (30 s), and stained in Meyer's hematoxylin (7 μM; 1 min). Following hematoxylin staining of nuclei, the slices were washed twice in DEPC water (30 s), dehydrated in a series of EtOH concentrations (i.e., EtOH 70%, EtOH 80%, EtOH 90%, twice EtOH 100%; 30 s in each), and dried on air (15 min). The laser parameters used for the microdissection were the following: Power 60, Aperture 7, Speed 7, Specimen Balance 46, and Offset 25. The pencil function was used to border the photoreceptor layers. Microdissected layers from each eye were pooled, and RNA was extracted, as described above.

miRNA Expression Analysis

qRT-PCR-based detection of mature miR-204, miR-124, and small nucleolar RNA 234 (sno234) was performed using the TaqMan miRNA assays (Applied Biosystems, Foster City, CA, USA). All reactions were performed in triplicate. The qRT-PCR results, recorded as threshold cycle numbers (Ct), were normalized to the sno234 reference, and the relative fold change of expression was calculated using the 2^{-ddCt} method.

SUPPLEMENTAL INFORMATION

Supplemental Information can be found online at https://doi.org/10.1016/j.omtn.2019.11.005.

AUTHOR CONTRIBUTIONS

M.K., E.M.S., and S.B. conceived the study, designed the experiments, analyzed the data, and wrote the manuscript. M.K., I.G., E.M., M.P., I.C., and E.M.S. carried out experimental work. R.D.C. and A.C. analyzed transcriptomic data. E.M. and E.M.S. performed subretinal injection and visual tests in mice. S.C. provided critical expertise for studies in the mouse. S.B. and E.M.S. provided funding for this study. All authors discussed the results and had the opportunity to comment on the manuscript.

CONFLICTS OF INTEREST

The authors declare no competing interests.

ACKNOWLEDGMENTS

The authors are grateful to Edoardo Nusco (TIGEM Animal Facility), Manuela Dionisi (TIGEM Next Generation Sequencing Facility), and Margherita Mutarelli (TIGEM Bioinformatics Core) for technical assistance. We would like to thank Alberto Auricchio and Brunella Franco for critical reading of the manuscript and Cathal Wilson for advice. This work was supported by the Foundation Fighting Blindness (TA-NMT-0616-0701-TIGM to S.B.), Fondazione Telethon (TGM16YGM02 to S.B.), and European Research Council (311682 "Allelechoker" to E.M.S.).

REFERENCES

1. Roska, B., and Sahel, J.A. (2018). Restoring vision. Nature *557*, 359–367.
2. Trapani, I., and Auricchio, A. (2018). Seeing the Light after 25 Years of Retinal Gene Therapy. Trends Mol. Med. *24*, 669–681.
3. Apte, R.S. (2018). Gene Therapy for Retinal Degeneration. Cell *173*, 5.
4. Ledford, H. (2017). FDA advisers back gene therapy for rare form of blindness. Nature *550*, 314.
5. Bartel, D.P. (2018). Metazoan MicroRNAs. Cell *173*, 20–51.
6. Karali, M., and Banfi, S. (2019). Non-coding RNAs in retinal development and function. Hum. Genet. *138*, 957–971.
7. Rupaimoole, R., and Slack, F.J. (2017). MicroRNA therapeutics: towards a new era for the management of cancer and other diseases. Nat. Rev. Drug Discov. *16*, 203–222.
8. Karali, M., Peluso, I., Marigo, V., and Banfi, S. (2007). Identification and characterization of microRNAs expressed in the mouse eye. Invest. Ophthalmol. Vis. Sci. *48*, 509–515.
9. Karali, M., Persico, M., Mutarelli, M., Carissimo, A., Pizzo, M., Singh Marwah, V., Ambrosio, C., Pinelli, M., Carrella, D., Ferrari, S., et al. (2016). High-resolution analysis of the human retina miRNome reveals isomiR variations and novel microRNAs. Nucleic Acids Res. *44*, 1525–1540.
10. Conte, I., Carrella, S., Avellino, R., Karali, M., Marco-Ferreres, R., Bovolenta, P., and Banfi, S. (2010). miR-204 is required for lens and retinal development via Meis2 targeting. Proc. Natl. Acad. Sci. USA *107*, 15491–15496.
11. Karali, M., Peluso, I., Gennarino, V.A., Bilio, M., Verde, R., Lago, G., Dollé, P., and Banfi, S. (2010). miRNeye: a microRNA expression atlas of the mouse eye. BMC Genomics *11*, 715.
12. Conte, I., Hadfield, K.D., Barbato, S., Carrella, S., Pizzo, M., Bhat, R.S., Carissimo, A., Karali, M., Porter, L.F., Urquhart, J., et al. (2015). MiR-204 is responsible for inherited retinal dystrophy associated with ocular coloboma. Proc. Natl. Acad. Sci. USA *112*, E3236–E3245.
13. Conte, I., Merella, S., Garcia-Manteiga, J.M., Migliore, C., Lazarevic, D., Carrella, S., Marco-Ferreres, R., Avellino, R., Davidson, N.P., Emmett, W., et al. (2014). The combination of transcriptomics and informatics identifies pathways targeted by miR-204 during neurogenesis and axon guidance. Nucleic Acids Res. *42*, 7793–7806.
14. Grant, C.A., Ponnazhagan, S., Wang, X.S., Srivastava, A., and Li, T. (1997). Evaluation of recombinant adeno-associated virus as a gene transfer vector for the retina. Curr. Eye Res. *16*, 949–956.
15. Allocca, M., Mussolino, C., Garcia-Hoyos, M., Sanges, D., Iodice, C., Petrillo, M., Vandenberghe, L.H., Wilson, J.M., Marigo, V., Surace, E.M., and Auricchio, A. (2007). Novel adeno-associated virus serotypes efficiently transduce murine photoreceptors. J. Virol. *81*, 11372–11380.
16. Li, T., Snyder, W.K., Olsson, J.E., and Dryja, T.P. (1996). Transgenic mice carrying the dominant rhodopsin mutation P347S: evidence for defective vectorial transport of rhodopsin to the outer segments. Proc. Natl. Acad. Sci. USA *93*, 14176–14181.
17. Groh, J., Ribechini, E., Stadler, D., Schilling, T., Lutz, M.B., and Martini, R. (2016). Sialoadhesin promotes neuroinflammation-related disease progression in two mouse models of CLN disease. Glia *64*, 792–809.
18. Liston, P., Fong, W.G., Kelly, N.L., Toji, S., Miyazaki, T., Conte, D., Tamai, K., Craig, C.G., McBurney, M.W., and Korneluk, R.G. (2001). Identification of XAF1 as an antagonist of XIAP anti-Caspase activity. Nat. Cell Biol. *3*, 128–133.
19. Leonard, K.C., Petrin, D., Coupland, S.G., Baker, A.N., Leonard, B.C., LaCasse, E.C., Hauswirth, W.W., Korneluk, R.G., and Tsilfidis, C. (2007). XIAP protection of photoreceptors in animal models of retinitis pigmentosa. PLoS ONE *2*, e314.
20. Wassmer, S.J., Leonard, B.C., Coupland, S.G., Baker, A.N., Hamilton, J., Hauswirth, W.W., and Tsilfidis, C. (2017). Overexpression of the X-Linked Inhibitor of Apoptosis Protects Against Retinal Degeneration in a Feline Model of Retinal Detachment. Hum. Gene Ther. *28*, 482–492.
21. Flannery, J.G., Zolotukhin, S., Vaquero, M.I., LaVail, M.M., Muzyczka, N., and Hauswirth, W.W. (1997). Efficient photoreceptor-targeted gene expression in vivo by recombinant adeno-associated virus. Proc. Natl. Acad. Sci. USA *94*, 6916–6921.
22. Ramamurthy, V., Niemi, G.A., Reh, T.A., and Hurley, J.B. (2004). Leber congenital amaurosis linked to AIPL1: a mouse model reveals destabilization of cGMP phosphodiesterase. Proc. Natl. Acad. Sci. USA *101*, 13897–13902.
23. Sohocki, M.M., Perrault, I., Leroy, B.P., Payne, A.M., Dharmaraj, S., Bhattacharya, S.S., Kaplan, J., Maumenee, I.H., Koenekoop, R., Meire, F.M., et al. (2000). Prevalence of AIPL1 mutations in inherited retinal degenerative disease. Mol. Genet. Metab. *70*, 142–150.

24. Liu, X., Bulgakov, O.V., Wen, X.H., Woodruff, M.L., Pawlyk, B., Yang, J., Fain, G.L., Sandberg, M.A., Makino, C.L., and Li, T. (2004). AIPL1, the protein that is defective in Leber congenital amaurosis, is essential for the biosynthesis of retinal rod cGMP phosphodiesterase. Proc. Natl. Acad. Sci. USA *101*, 13903–13908.

25. Allocca, M., Manfredi, A., Iodice, C., Di Vicino, U., and Auricchio, A. (2011). AAV-mediated gene replacement, either alone or in combination with physical and pharmacological agents, results in partial and transient protection from photoreceptor degeneration associated with betaPDE deficiency. Invest. Ophthalmol. Vis. Sci. *52*, 5713–5719.

26. Deveraux, Q.L., Takahashi, R., Salvesen, G.S., and Reed, J.C. (1997). X-linked IAP is a direct inhibitor of cell-death proteases. Nature *388*, 300–304.

27. Latour, S., and Aguilar, C. (2015). XIAP deficiency syndrome in humans. Semin. Cell Dev. Biol. *39*, 115–123.

28. Yao, J., Feathers, K.L., Khanna, H., Thompson, D., Tsilfidis, C., Hauswirth, W.W., Heckenlively, J.R., Swaroop, A., and Zacks, D.N. (2011). XIAP therapy increases survival of transplanted rod precursors in a degenerating host retina. Invest. Ophthalmol. Vis. Sci. *52*, 1567–1572.

29. Brown, G.C., and Neher, J.J. (2014). Microglial phagocytosis of live neurons. Nat. Rev. Neurosci. *15*, 209–216.

30. Cuenca, N., Fernández-Sánchez, L., Campello, L., Maneu, V., De la Villa, P., Lax, P., and Pinilla, I. (2014). Cellular responses following retinal injuries and therapeutic approaches for neurodegenerative diseases. Prog. Retin. Eye Res. *43*, 17–75.

31. Zhao, L., Zabel, M.K., Wang, X., Ma, W., Shah, P., Fariss, R.N., Qian, H., Parkhurst, C.N., Gan, W.B., and Wong, W.T. (2015). Microglial phagocytosis of living photoreceptors contributes to inherited retinal degeneration. EMBO Mol. Med. *7*, 1179–1197.

32. Rashid, K., Wolf, A., and Langmann, T. (2018). Microglia Activation and Immunomodulatory Therapies for Retinal Degenerations. Front. Cell. Neurosci. *12*, 176.

33. Hudry, E., and Vandenberghe, L.H. (2019). Therapeutic AAV Gene Transfer to the Nervous System: A Clinical Reality. Neuron *101*, 839–862.

34. Hildinger, M., Auricchio, A., Gao, G., Wang, L., Chirmule, N., and Wilson, J.M. (2001). Hybrid vectors based on adeno-associated virus serotypes 2 and 5 for muscle-directed gene transfer. J. Virol. *75*, 6199–6203.

35. Liang, F.Q., Anand, V., Maguire, A.M., and Bennett, J. (2001). Intraocular delivery of recombinant virus. Methods Mol. Med. *47*, 125–139.

36. Barbato, S., Marrocco, E., Intartaglia, D., Pizzo, M., Asteriti, S., Naso, F., Falanga, D., Bhat, R.S., Meola, N., Carissimo, A., et al. (2017). MiR-211 is essential for adult cone photoreceptor maintenance and visual function. Sci. Rep. *7*, 17004.

37. Botta, S., de Prisco, N., Marrocco, E., Renda, M., Sofia, M., Curion, F., Bacci, M.L., Ventrella, D., Wilson, C., Gesualdo, C., et al. (2017). Targeting and silencing of rhodopsin by ectopic expression of the transcription factor KLF15. JCI Insight *2*, e96560.

38. Pinelli, M., Carissimo, A., Cutillo, L., Lai, C.H., Mutarelli, M., Moretti, M.N., Singh, M.V., Karali, M., Carrella, D., Pizzo, M., et al. (2016). An atlas of gene expression and gene co-regulation in the human retina. Nucleic Acids Res. *44*, 5773–5784.

39. Huang, W., Sherman, B.T., and Lempicki, R.A. (2009). Systematic and integrative analysis of large gene lists using DAVID bioinformatics resources. Nat. Protoc. *4*, 44–57.

40. Paraskevopoulou, M.D., Georgakilas, G., Kostoulas, N., Vlachos, I.S., Vergoulis, T., Reczko, M., Filippidis, C., Dalamagas, T., and Hatzigeorgiou, A.G. (2013). DIANA-microT web server v5.0: service integration into miRNA functional analysis workflows. Nucleic Acids Res. *41*, W169–W173.

41. Lewis, B.P., Burge, C.B., and Bartel, D.P. (2005). Conserved seed pairing, often flanked by adenosines, indicates that thousands of human genes are microRNA targets. Cell *120*, 15–20.

42. Gennarino, V.A., Sardiello, M., Avellino, R., Meola, N., Maselli, V., Anand, S., Cutillo, L., Ballabio, A., and Banfi, S. (2009). MicroRNA target prediction by expression analysis of host genes. Genome Res. *19*, 481–490.

OMTN, Volume *19*

Supplemental Information

AAV-miR-204 Protects from Retinal Degeneration by Attenuation of Microglia Activation and Photoreceptor Cell Death

Marianthi Karali, Irene Guadagnino, Elena Marrocco, Rossella De Cegli, Annamaria Carissimo, Mariateresa Pizzo, Simona Casarosa, Ivan Conte, Enrico Maria Surace, and Sandro Banfi

Figure S1

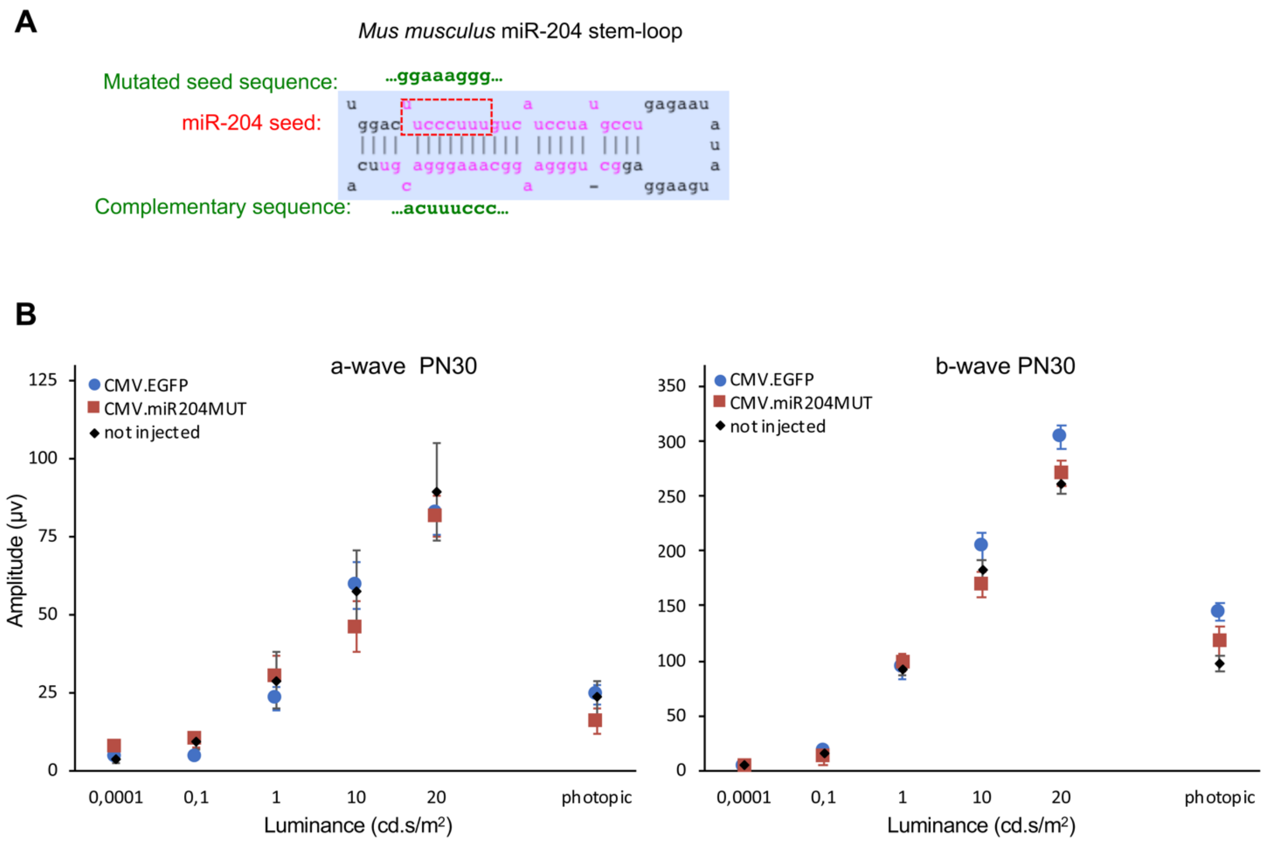

Figure S1. ERG analysis of *RHO*-P347S transgenic mice following AAV-mediated delivery of control vectors used in this study at PN4.

(A) Sequence of the miR-204 stem-loop in *Mus musculus* (blue box). The sequence of the mature miR-204-5p (upper strand) and miR-204-3p (lower strand) is in purple. The 'seed' region of miR-204-5p is in a red frame. The sequence introduced by site-directed mutagenesis to generate the AAV.CMV.miR204MUT control vector is in green.

(B) A-wave and b-wave ERG responses of *RHO*-P347S mice injected with AAV.CMV.EGFP (n=26) and AAV.CMV.miR204MUT (n=9) at PN4. Responses of non-injected eyes are shown in black. ERGs were recorded at PN30. Both control vectors elicited similar ERG responses and did not show significant differences compared to non-injected *RHO*-P347S eyes.

Figure S2

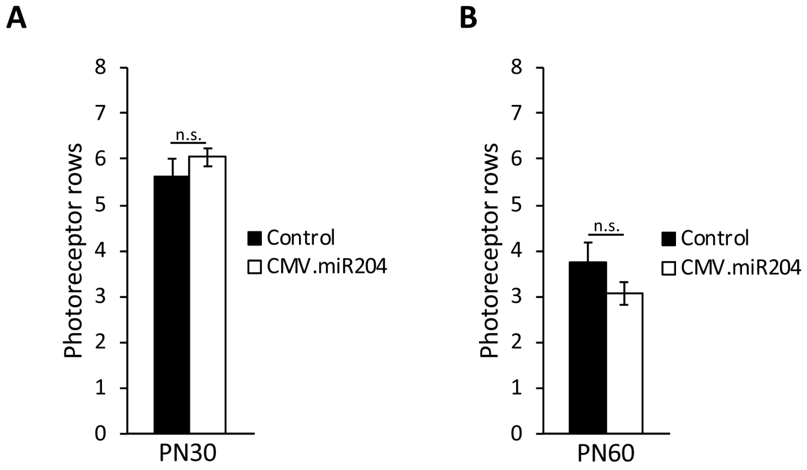

Figure S2. ONL thickness in injected *RHO*-P347S eyes.
Plots show the average number of photoreceptor nuclei rows at PN30 **(A)** and PN60 **(B)** in miR-204 treated *RHO*-P347S eyes (CMV.miR204) compared to contralateral eyes injected with the control vector (CMV.EGFP) at PN4 (n=4 animals per stage; Error bars are SEM; n.s.: not significant). Statistical significance (PN30, p-value=0.76; PN60, p-value=0.67) was calculated with a GLM analysis. For each animal, at least 6 retinal sections were analysed and photoreceptor rows were counted in three different parts of each section, distributed within the transduced areas (dorsal eye).

Figure S3

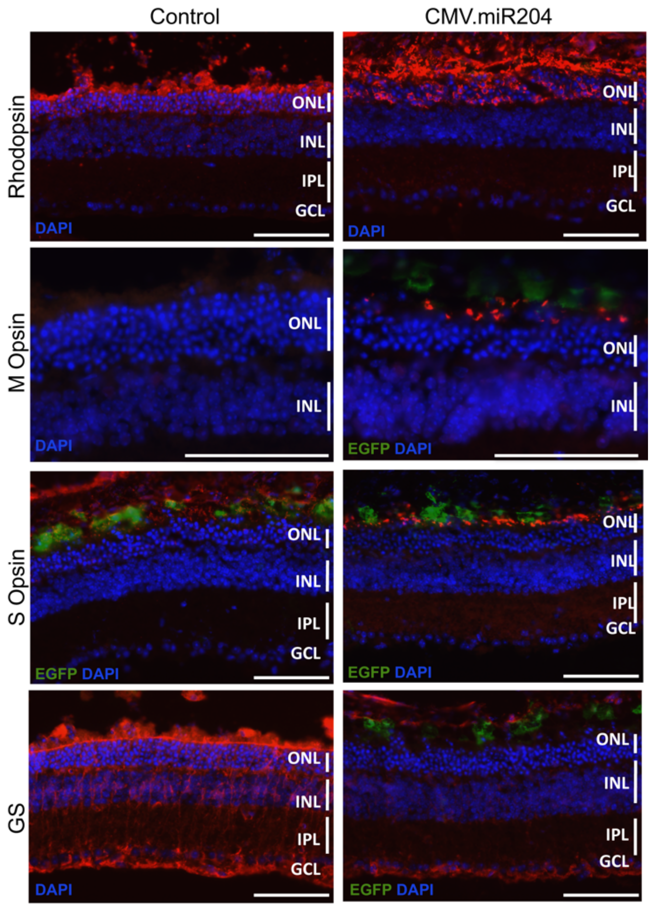

Figure S3. Expression studies on retinas from 2-month old *RHO*-P347S transgenic mice following AAV-mediated delivery of miR-204 at PN4.

Immunolabeling for cone photoreceptor markers (M Opsin, S Opsin; in red) and Glutamine Synthetase (GS; in red) on PN60 retinal sections. Nuclei were counterstained with DAPI (blue). Control virus distribution is shown by the EGFP fluorescence (green). For markers with polarised expression (e.g. M Opsin, S Opsin) comparisons were made between corresponding dorsal and ventral areas. Scale bar: 75 µm.

Abbreviations: GCL, ganglion cell layer; INL, inner nuclear layer; ONL, outer nuclear layer; RPE, Retinal Pigment Epithelium.

Figure S4

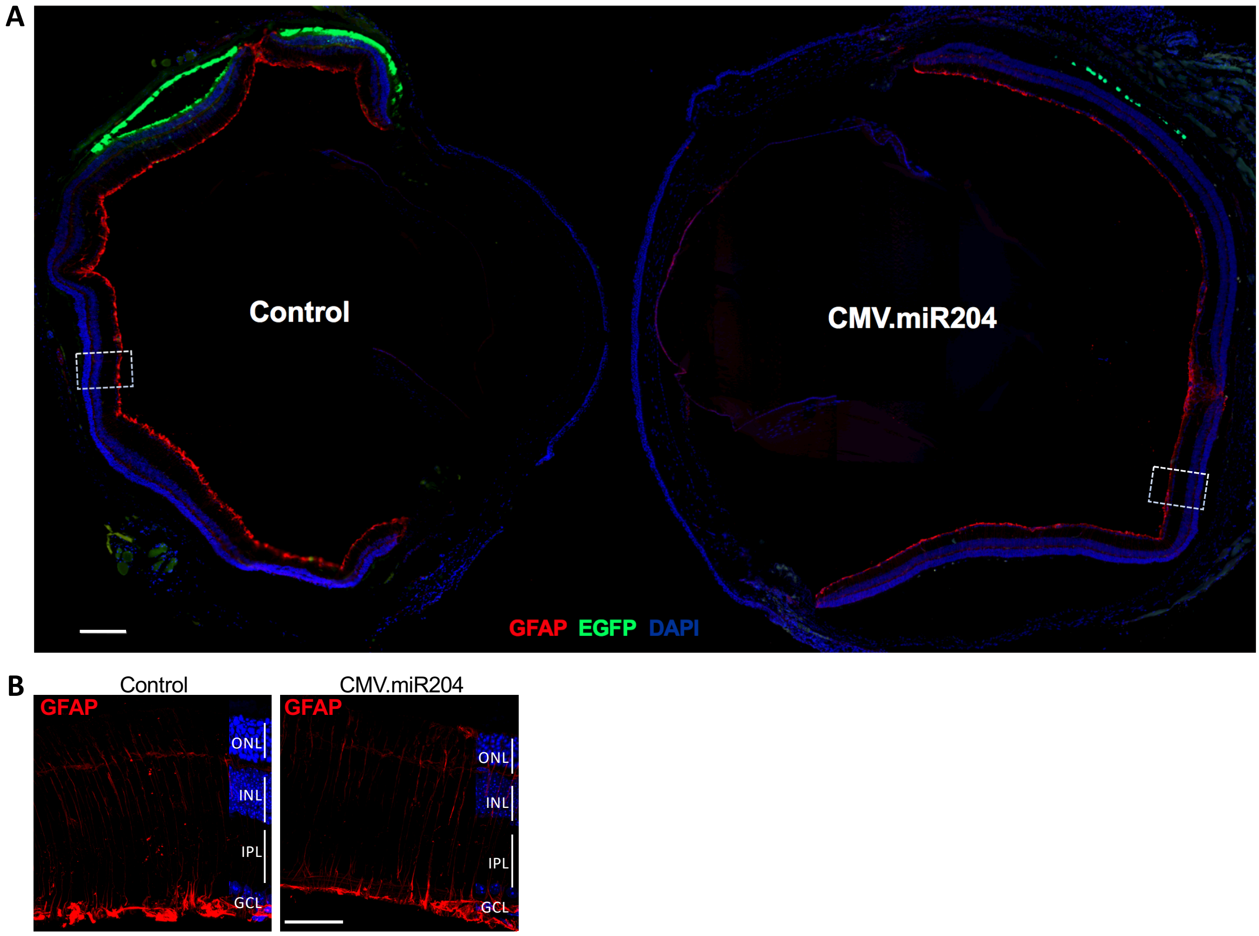

Figure S4. AAV-mediated delivery of miR-204 at PN4 reduces reactive gliosis in *RHO-P347S* mice.
(A) Panoramic view of entire retina sections at PN30 immunolabelled for Glial Fibrillary Acidic Protein (GFAP; in red). Nuclei were counterstained with DAPI (blue). Control virus delivery is visible by the EGFP fluorescence (green) and is more evident in the RPE of the control-treated eye (left-hand side) injected with the AAV.CMV.EGFP vector ($1.1x10^9$ GC). The contralateral eye was injected with the AAV.CMV.miR204 vector mix ($1x10^9$ GC AAV.CMV.miR204, $1x10^8$ GC AAV.CMV.EGFP). Both sections were present on the same slide and images were acquired as orthogonal projections at the Zeiss Axio Scan.Z1 (20X magnification; Z-stack, 8 slices, 1 μm intervals). The CMV.miR204-treated eye shows an apparent decrease in retinal gliosis, a physiological response to PR damage, compared to the contralateral control-injected eye. Scale bar: 200 μm.
(B) Confocal microscope images (63X magnification) from the boxed areas in (A). Immunolabeling for GFAP is shown in red. Scale bar: 50 μm.
Abbreviations: GCL, ganglion cell layer; INL, inner nuclear layer; IPL, inner plexiform layer; ONL, outer nuclear layer.

Figure S5

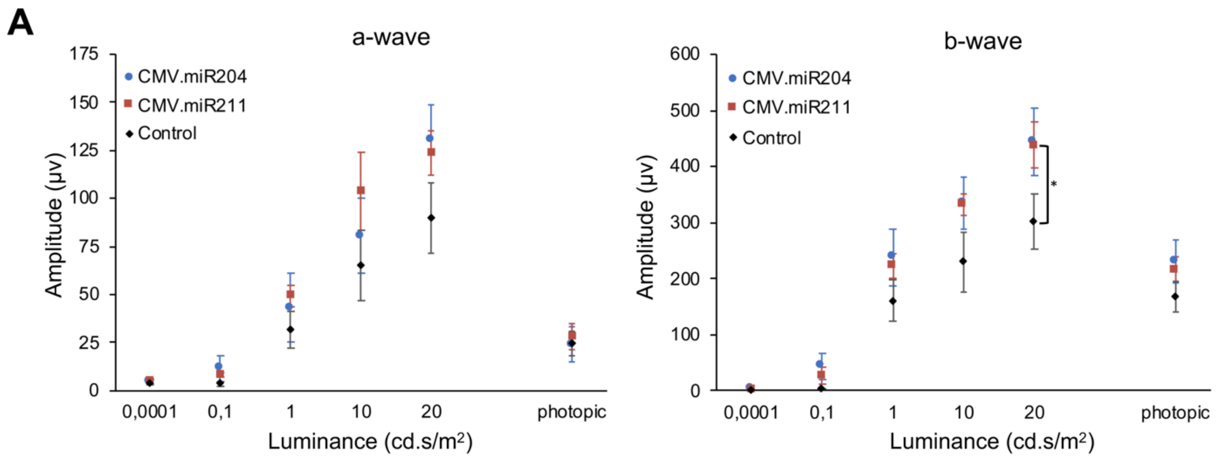

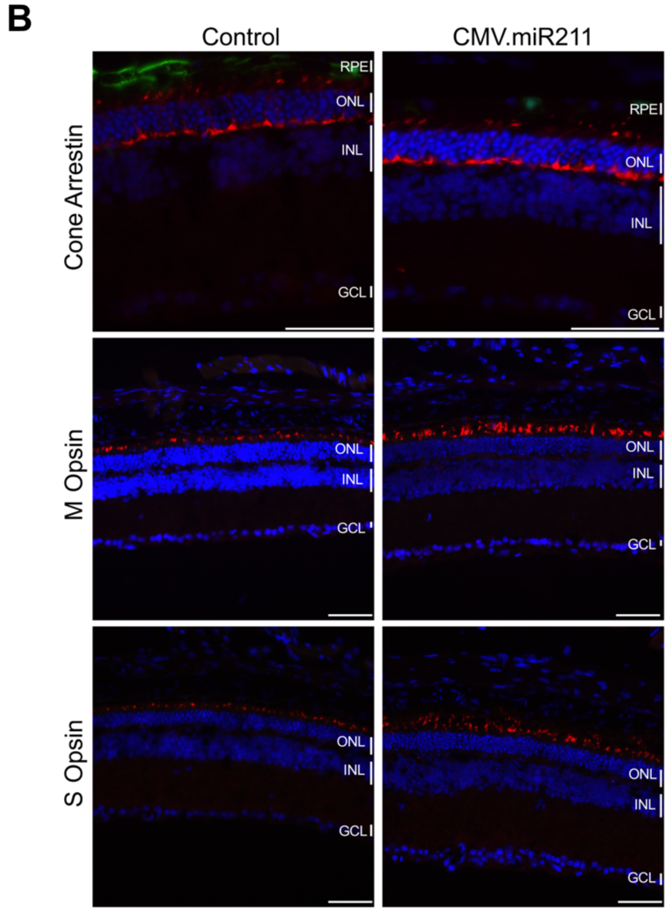

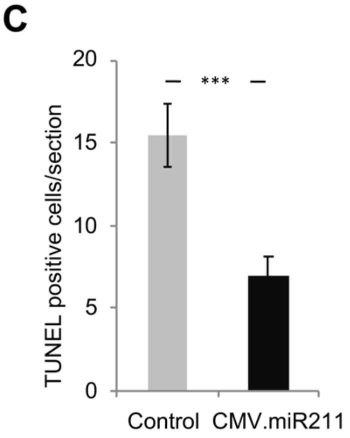

Figure S5. Functional and molecular analysis of *RHO*-P347S transgenic mice following AAV-mediated delivery of miR-211 at PN4.

(A) A-wave and b-wave ERG responses recorded at PN30 from *RHO*-P347S mice injected with AAV.CMV.miR211, AAV.CMV.miR204 and control vectors at PN4.

(B) Immunolabeling for cone photoreceptor markers (M Opsin, S Opsin, Cone Arrestin; in red) on retinal sections from *RHO*-P347S eyes injected with AAV.CMV.miR211. Nuclei were counterstained with DAPI (blue). Control virus distribution (co-injected at a 1:10 ratio with AAV.CMV.miR211) is shown by the EGFP fluorescence (green). Cone opsin expression was better preserved in miR211-treated eyes. Abbreviations: GCL, ganglion cell layer; INL, inner nuclear layer; ONL, outer nuclear layer; RPE, Retinal Pigment Epithelium. Scale bar: 50 µm.

(C) TUNEL-positive nuclei at the ONL of eyes injected with the AAV.CMV.miR211 and control vectors. Corresponding sections from serially-sectioned eyes were counted. The mean number of apoptotic photoreceptor cells was significantly reduced in the retina of *RHO*-P347S mice following injection of AAV.CMV.miR211 compared to contralateral control-treated eyes. Error bars are SEM. n=2 retinas. Statistical significance (p-value=0.0001736) was calculated with a GLM analysis.

Figure S6

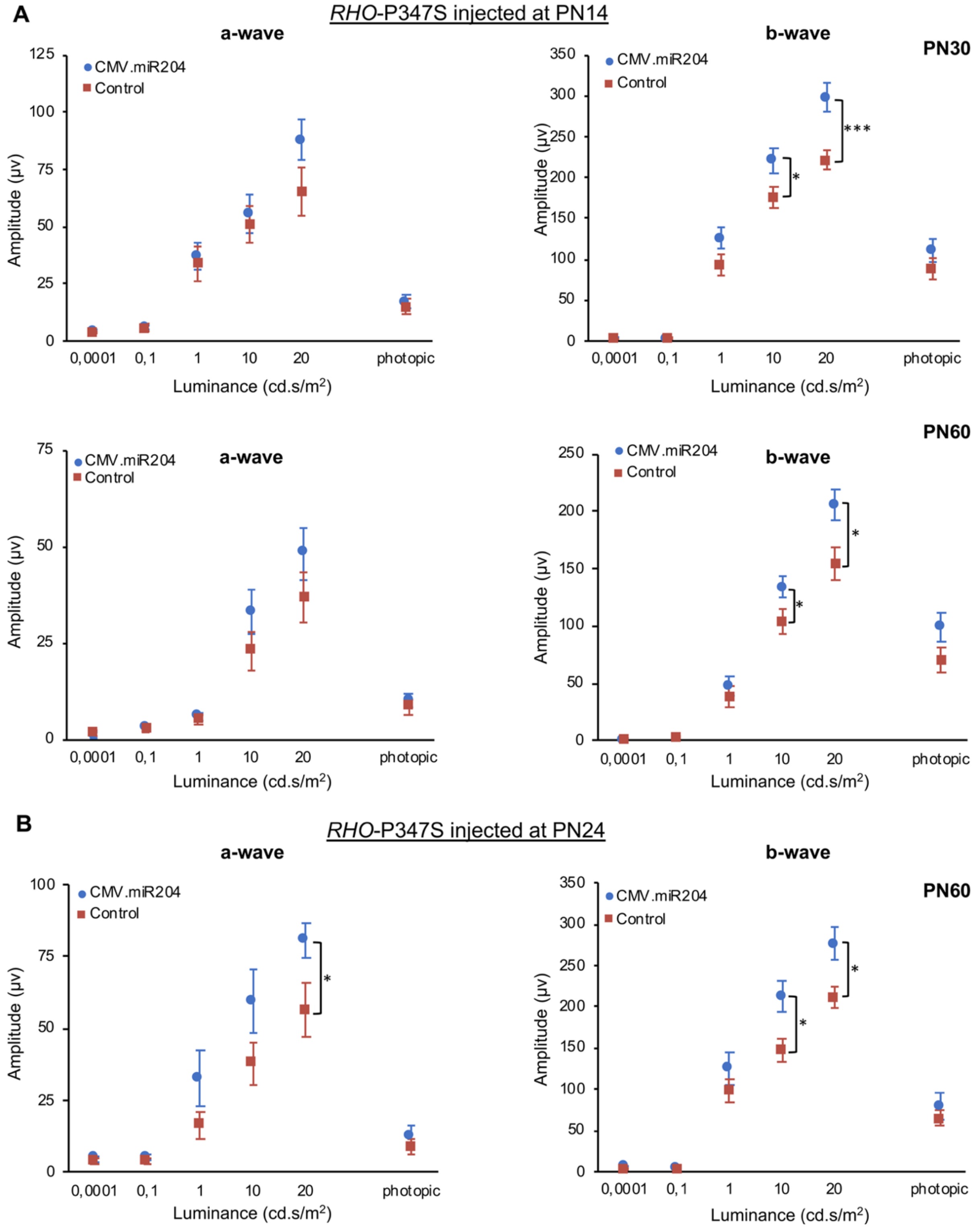

Figure S6. Protective effect of miR-204 injection at PN14 and PN24 in *RHO*-P347S mice.
(A) ERG responses of *RHO*-P347S mice injected with AAV.CMV.miR204 and the control vector at PN14. ERGs were recorded at PN30 (n=18) and PN60 (n=13). Subretinal injection of AAV.CMV.miR204 at PN14 results in an improvement of retinal function at PN30 and PN60, indicated by a statistically significant increase in b-wave amplitudes vs contralateral eyes injected with the control vector.
(B) A- and b-wave ERG responses of *RHO*-P347S mice (n=11) injected with AAV.CMV.miR204 and control vector at PN24. ERGs were recorded at PN60.
Viral doses were: $1x10^9$ GC AAV.CMV.miR204, $1x10^8$ GC AAV.CMV.EGFP per eye; $1x10^9$ GC AAV.CMV.miR204MUT, $1x10^8$ GC AAV.CMV.EGFP per eye. Statistical significance (unpaired Student's t-test against control vector) is indicated with asterisks (*p<0.05, ***p<0.001).

Figure S7

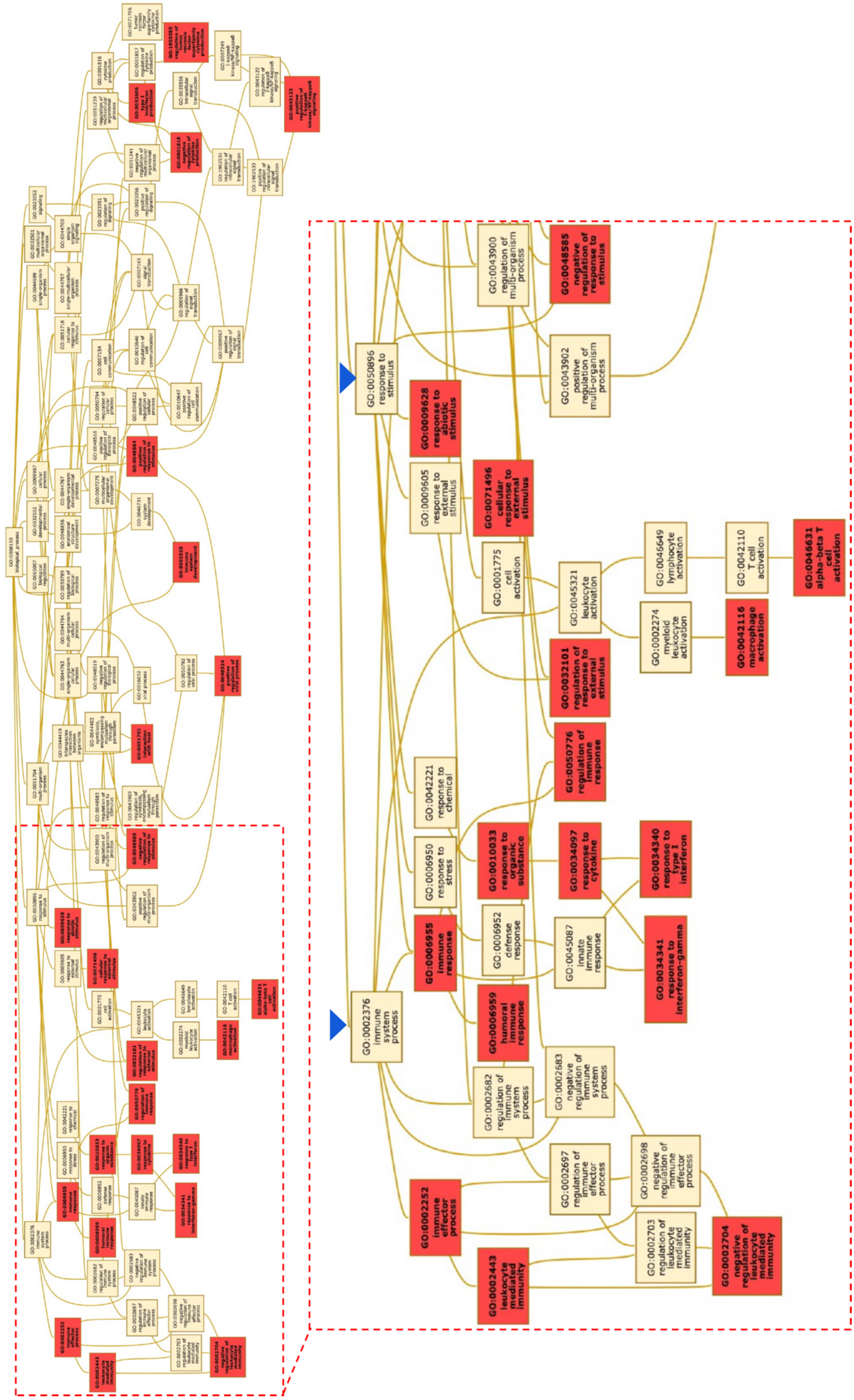

(see legend in the following page)

Figure S7. GO-view visualisation of BP GO-terms enriched among DEGs downregulated following miR-204 delivery in *RHO*-P347S retinas.

The graph shows the relationship between the GO terms based on Biological Processes (BPs) and their ancestor terms: 24 clusters (out of 58, red boxes) enriched among the downregulated genes in a statistically significant manner are directly related to the ancestor GO term 'immune system process' (GO:0002376, blue arrowhead). Terms were also related to the 'response to stimulus' (GO:0050896, blue arrowhead) which is a 'parent-term' of the inflammatory response-related terms.

Figure S8

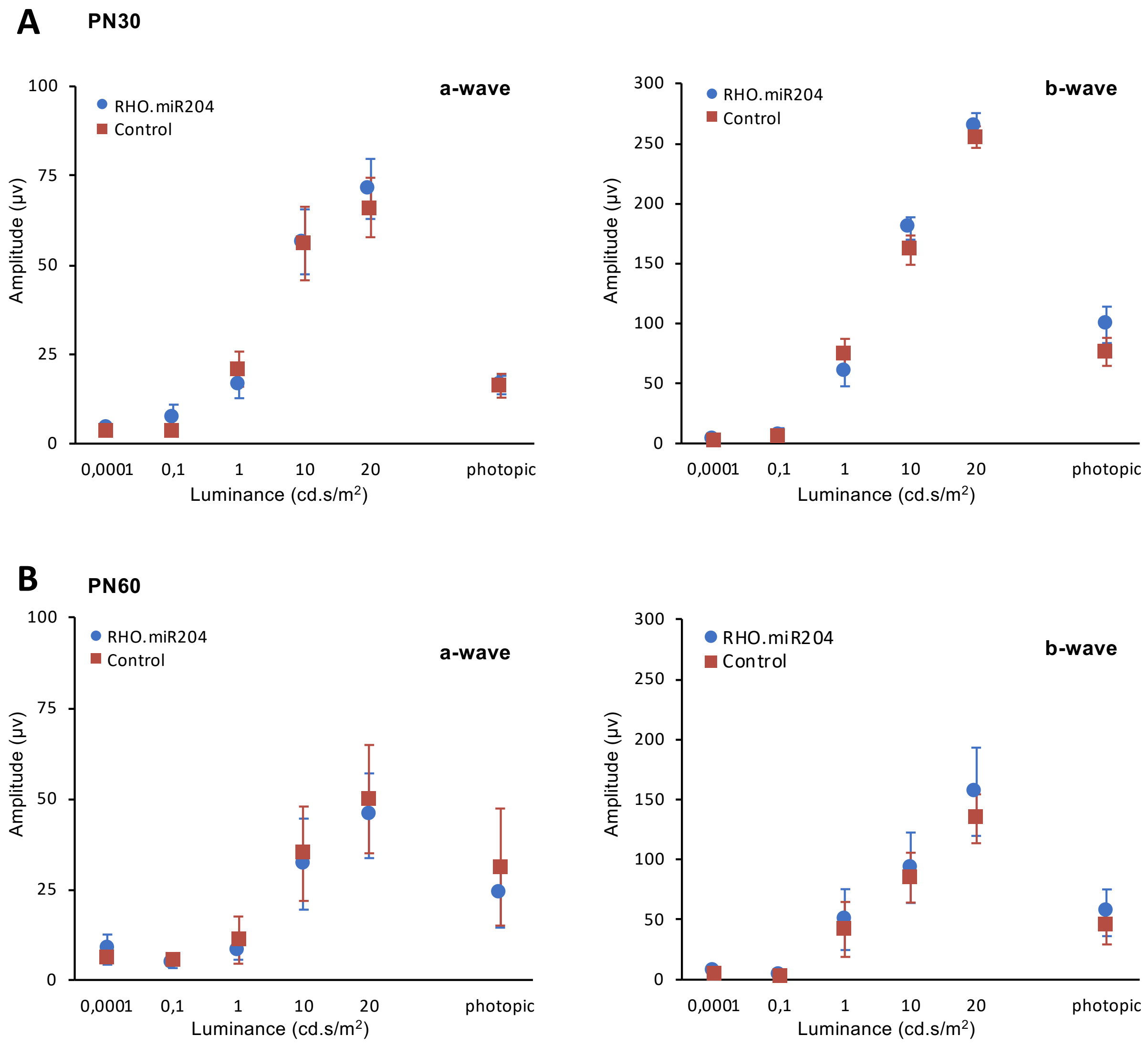

Figure S8. AAV-mediated delivery of miR-204 using a photoreceptor-specific promoter at PN14 does not improve ERG responses in *RHO*-P347S mice.
ERG responses of *RHO*-P347S mice injected with the AAV.RHO.miR204 and control vector (AAV.RHO.EGFP) at PN14. ERGs were recorded at PN30 (n=17; panel A) and PN60 (n=5; panel B). No statistically significant improvement of a- and b-wave amplitudes was recorded at PN30 and PN60 in RHO.miR204-treated vs contralateral control eyes. Vector doses were: 1x10^9 GC AAV.RHO.miR204, 1x10^8 GC AAV.RHO.EGFP per eye; 1.1x10^9 GC AAV.RHO.EGFP per control eye.

Supplemental Tables

(see separate Excel files)

Table S1. Normalised read counts of the differentially expressed genes between retinas of wild-type c57BL/6 mice injected with the AAV.CMV.miR204 and control AAV.CMV.miR204MUT vector.
Ensemble ID and Gene Symbol of Differentially Expressed (FDR<0.1) genes between retinas from c57BL/6 mice injected with the AAV.CMV.miR204 (columns c57.Bl6_animalX_OS_CMV.miR204) and AAV.CMV.miR204MUT vector (columns c57.Bl6_animalX_OD_CMV.miR204MUT) in a contralateral manner. Downregulated genes (n=103) are highlighted in green, upregulated genes (n=49) in red. Abbreviations: logFC: log fold change, logCPM: log counts per million, LR: likelihood ratio, p-value: Probability value, FDR: False Discovery Rate.

Table S2. Gene Ontology enrichment analysis of the differentially expressed genes (DEGs) between retinas of C57BL/6 mice injected with the AAV.CMV.miR204 and control AAV.CMV.miR204MUT vector.
The Biological Process (BP_FAT) and Cellular Component (CC_FAT) terms enriched among the down-regulated (n=103) and up-regulated (n=49) genes are highlighted in green and red, respectively. For each BP/CC annotation cluster, the GO identifier ("GO:"), associated term ("Term") and Enrichment score are provided. The genes belonging to each term are listed in column D (and their total number in column C under “count”). False Discovery Rate score (FDR) is shown in column E.

Table S3. Normalised read counts of the differentially expressed genes between retinas of *RHO*-P347S mice (n=3) injected with the AAV.CMV.miR204 and control AAV.CMV.miR204MUT vector.
Ensemble ID and Gene Symbol of differentially expressed genes (FDR<0.1) between retinas from *RHO*-P347S mice injected with the AAV.CMV.miR204 (columns c57.Bl6_animalX_OS_CMV.miR204) and AAV.CMV.miR204MUT vector (columns c57.Bl6_animalX_OD_CMV.miR204MUT) in a contralateral manner. Downregulated genes (n=76) are highlighted in green, upregulated genes (n=7) in red. Abbreviations: logFC: log fold change, logCPM: log counts per million, LR: likelihood ratio, p-value: Probability value, FDR: False Discovery Rate.

Table S4. Gene Ontology enrichment analysis of the differentially expressed genes (DEGs) between retinas of *RHO*-P347S mice (n=3) injected with the AAV.CMV.miR204 and control AAV.CMV.miR204MUT vector.
The Biological Process (BP_FAT) and Cellular Component (CC_FAT) terms enriched among the downregulated (n=76) genes are highlighted in green. For each BP/CC annotation cluster, the GO identifier ("GO:"), associated term ("Term") and Enrichment score are provided. The genes belonging to each term are listed in column D (and their total number in column C under “count”). False Discovery Rate score (FDR) is shown in column E. The small number of up-regulated genes (n=7) precluded a corresponding GO enrichment analysis.

Table S5. Normalised read counts of the differentially expressed genes between retinas of *RHO*-P347S mice (n=2) injected with the AAV.CMV.miR204 and control AAV.CMV.miR204MUT vector.
Ensemble ID and Gene Symbol of differentially expressed genes (FDR<0.1) between retinas from *RHO*-P347S mice (animal number 2 and 5) injected with the AAV.CMV.miR204 (columns *RHO*-P347S_animalX_OS_CMV.miR204) and AAV.CMV.miR204MUT vector (columns *RHO*-P347S_animalX_OD_CMV.miR204MUT) in a contralateral manner. Downregulated genes (n=316) are highlighted in green, upregulated genes (n=104) in red.
Abbreviations: logFC: log fold change, logCPM: log counts per million, LR: likelihood ratio, p-value: Probability value, FDR: False Discovery Rate.

Table S6. Gene Ontology enrichment analysis of the differentially expressed genes (DEGs) between retinas of *RHO*-P347S mice (n=2) injected with the AAV.CMV.miR204 and control AAV.CMV.miR204MUT vector.
The Biological Process (BP_FAT) and Cellular Component (CC_FAT) terms enriched among DEGs (n=420) are highlighted in green for the downregulated (n=316) and in red for the upregulated genes (n=104). For each BP/CC annotation cluster, the GO identifier ("GO:"), associated term ("Term") and Enrichment score are provided. The genes belonging to each term are listed in column D (and their total number in column C under “count”). False Discovery Rate score (FDR) is shown in column E.

Table S7. Normalised read counts of the differentially expressed genes (DEGs) between retinas of c57BL/6 (n=3) and *RHO*-P347S mice (n=2) injected with the control vector AAV.CMV.miR204MUT.
Ensemble ID and Gene Symbol of differentially expressed genes (FDR<0.1) between control-injected retinas from c57BL/6 (columns c57.Bl6_animalX_OD_CMV.miR204MUT) and RHO-P347S mice (columns RHO-P347S _animalX_OD_CMV.miR204MUT). Downregulated genes (n=2158) are highlighted in green, upregulated genes (n=2053) in red. Abbreviations: logFC: log fold change, logCPM: log counts per million, LR: likelihood ratio, p-value: Probability value, FDR: False Discovery Rate.

Table S8. Gene Ontology enrichment analysis of the differentially expressed genes (DEGs) between retinas of c57BL/6 (n=3) and *RHO*-P347S mice (n=2) injected with the control vector AAV.CMV.miR204MUT.

The Biological Process (BP_FAT) and Cellular Component (CC_FAT) terms enriched among DEGs (n=4211) are highlighted in green for the downregulated (n=2158) and in red for the upregulated genes (n=2053). For each BP/CC annotation cluster, the GO identifier ("GO:"), associated term ("Term") and Enrichment score are provided. The genes belonging to each term are listed in column D (and their total number in column C under “count”). False Discovery Rate score (FDR) is shown in column E.

Table S9. List of differentially expressed genes (DEGs; n=290) that show the same trend of down- or upregulation both upon miR204 delivery in *RHO*-P347S mice (compared to delivery of the control vector) and in control-treated wild-type (c57BL/6) retinas (compared to control-treated *RHO*-P347S).
Genes that are significantly downregulated in both comparisons are highlighted in green. Upregulated genes are highlighted in red. Ensemble ID, Gene Symbol and normalised read counts are shown for DEGs with FDR<0.1. Abbreviations: logFC: log fold change, logCPM: log counts per million, LR: likelihood ratio, p-value: Probability value, FDR: False Discovery Rate.

Table S10. Gene Ontology enrichment analysis of the genes that are differentially expressed both upon miR204 delivery in *RHO*-P347S mice (compared to delivery of the control vector) and in control-treated wild-type (c57BL/6) retinas (compared to control-treated *RHO*-P347S).
The Biological Process (BP_FAT) and Cellular Component (CC_FAT) terms enriched among the common DEGs (n=290) are highlighted in green for DEGs downregulated in both comparisons and in red for the commonly upregulated ones. For each BP/CC annotation cluster, the GO identifier ("GO:"), associated term ("Term") and Enrichment score are provided. The genes belonging to each term are listed in column D (and their total number in column C under “count”). False Discovery Rate score (FDR) is shown in column E.
